# Mutation of the TYTLE Motif in the Cytoplasmic Tail of the Sendai Virus Fusion Protein Deeply Affects Viral Assembly and Particle Production

**DOI:** 10.1371/journal.pone.0078074

**Published:** 2013-12-10

**Authors:** Manel Essaidi-Laziosi, Anastasia Shevtsova, Denis Gerlier, Laurent Roux

**Affiliations:** 1 Department of Microbiology and Molecular Medicine, Faculty of Medicine, University of Geneva, Geneva, Switzerland; 2 Centre International de Recherche en Infectiologie, Université Lyon 1, ENS de Lyon, Lyon, France; University of Edinburgh, United Kingdom

## Abstract

Enveloped viruses contain glycoproteins protruding from the viral membrane. These proteins play a crucial role in the extra-cellular steps of the virus life cycle, namely attachment to and entry into cells. Their role during the intracellular late phase of virus multiplication has been less appreciated, overlooked by the documented central organizer role of the matrix M protein. Sendai virus, a member of the Paramyxoviridae family, expresses two trans-membrane proteins on its surface, HN and F. In previous work, we have shown that suppression of F in the context of an infection, results in about 70% reduction of virus particle production, a reduction similar to that observed upon suppression of the matrix M protein. Moreover, a TYTLE motif present in F cytoplasmic tail has been proposed essential for virus particle production. In the present work, using original alternate conditional siRNA suppression systems, we generated a double F gene recombinant Sendai virus expressing wt-F and a nonviable mutated TYTLE/5A F protein (F_5A_). Suppression of the wild type F gene expression in cells infected with this virus allowed the analysis of F_5A_ properties in the context of the infection. Coupling confocal imaging analysis to biochemical characterization, we found that F_5A_ i) was not expressed at the cell surface but restricted to the endoplasmic reticulum, ii) was still capable of interaction with M and iii) had profound effect on M and HN cellular distribution. On the basis of these data, we propose a model for SeV particle formation based on an M/F complex that would serve as nucleation site for virus particle assembly at the cell surface.

## Introduction

Enveloped viruses contain trans-membrane glycoproteins that protrude from the particle envelope. The glycoproteins also shortly extend from the inner side of the envelope to contact the matrix protein and/or the viral core. These so-called cytoplasmic tails (ct) vary in size, ranging from a few amino acid residues to several tens. Upon infection, these glycoproteins have a dual role. They allow attachment of the particle by binding to specific cellular receptors and they orchestrate the fusion of the viral envelope with the cellular membrane to deliver the viral genome inside the cell.

To these essential roles, the involvement of the glycoproteins in virus particle formation and production has also been recognized, namely their participation in the formation of the assembly complex at the membrane, as well as in the induction of the membrane curvature leading to the formation of the viral bud (pull effect, for a review on the subject see Welsch et al. [1]). In view of the well-established role of the matrix protein M described as the central organizer of the assembly complex, the implication of the glycoproteins in these late steps has gradually grown in importance, although there are variations depending on the virus. More specifically, and as examples, the HIV Env appears dispensable as Gag alone buds very efficiently [2] [3] [4] [5]. This contrasts with the 30 fold increased budding of rabies virus like particles (VLP) promoted by its glycoprotein G [6]. Similarly, the influenza virus hemagglutinin (HA) and neuraminidase (NA) have been recognized more compulsory for VLP production than the matrix protein M1 [7]. A critical role for the hemagglutinin-neuraminidase (HN) protein of the Parainfluenza virus type 5 (PIV5) was equally reported, emphasizing the involvement of its cytoplasmic tail domain at the expense of the M and, interestingly, of the other glycoprotein F 8–10.

Sendai virus (SeV), as influenza virus or PIV5, contains two glycoproteins protruding on its envelope, HN and F, with, respectively, receptor binding/cleavage and viral envelope/cellular membrane fusion activities. To evaluate the importance of these two surface glycoproteins in the process of particle production, recombinant Sendai viruses (rSeV) were produced with HN and F harboring gradual truncations of, as well as site directed mutations in their cytoplasmic tail [11]. For HN (type II glycoprotein, ct = 35 aa at the N-terminus), a motif _10_SYWST_14_, described previously as required for HN incorporation into virions [12], was confirmed in this function. However, production of normal level of virus particles with undetectable amount of HN was found possible, making HN dispensable for virus particle production. For F (type I glycoprotein, ct = 42 aa at the C-terminus), i) truncation of the C-ter 16 aa was allowed, ii) truncation of 38 aa reduced production of virus particle by 30–50 folds, iii) random mutation of aa 17–28 reduced virus particle production by more than 100 folds, and finally iv) the motif _20_TYTLE_24_ appeared to be compulsory for virus particle production [13]. While virus particles with low/no HN can be produced, no virus particle could be found with poor F contents, i.e. production of virus particle is always reduced upon F mutations, suggesting that F controls the level of virus particle production. These conclusions were confirmed and extended recently with the availability of new rSeV's open to surface protein conditional suppression by the siRNA technology [14]. The virus production drop seen upon F suppression parallels that seen upon M suppression [15], suggesting a similar requirement for F in the assembly process. Reassuringly, HN suppression by itself was of no consequence for virion production. Concomitant suppression of HN and F, however, provoked the most significant decrease of virus particle production, alluding to a partially redundant role of F and HN, in which F can fully complement for the absence of HN. At this point, F was predicted essential for virus particle production, as was the key TYTLE motif in its cytoplasmic tail.

We present here experiments that define how the F protein is fulfilling its essential role in SeV production. Using different approaches based on siRNA technology, involving integrated suppression complementation system (ISCS) as well as alternate conditional suppression (biG-biS, Denis Gerlier et al. manuscript submitted), a rSeV was engineered in the end that expresses a non-functional F protein harboring a TYTLE motif substituted with 5 alanines (TYTLE/5A, HA-F_5A_). Infections with this rSeV-expressing HA-F_5A_ produced no virus particles but allowed unraveling the role of this protein in virus particle formation. Biochemical and confocal image analysis reveal that HA-F_5A_ is not expressed at the cell surface as it remains stuck in the endoplasmic reticulum. As it is still capable of binding to M, the subcellular distribution of this latter is modified, resulting in the diminution of M at the inner surface of the plasma membrane. Concomitant with these changes, the HN expression pattern at the cell surface is altered. These observations support a model for viral assembly in which F bound to M would form a virion assembly nucleation site at the plasma membrane.

## Materials and Methods

### Cells and virus infection

MDCK and BSRT7 cells were grown at 37°C under 5% CO_2_ atmosphere in respectively DMEM (Dulbecco's Modified Eagle Medium, Gibco-Invitrogen) and GMEM (BHK-21 Glascow minimum essential medium, Gibco-Invitrogen) supplemented with 5% foetal calf serum (FCS, Brunshwing) and penicillin and streptomycin antibiotics (Gibco). BSRT7 cells obtained from K.K. Conzelmann (Max von Pettenkofer-Institute and Gene Center, Ludwig-Maximilians-University, Munich) were treated with 0.5 µg/ml of Geneticin (Gibco) once every two passages. MDCK cells expressing α-gfpt siRNA have been, respectively, described in detail before [15]. BSRT7 stably expressing α-mvpt siRNA and the puromycin N-acetyl-transferase were obtained by lentifection (Denis Gerlier et al. manuscript submitted) and treated with 1 µg/ml µg Puromycin (Sigma) once every two passages. For infection, virus was diluted in basic DMEM/GMEM and adsorbed onto cells during 1 hour at 33°C. At the end of the infection period, the infectious medium was replaced by 2% FCS-DMEM/GMEM. Cells and supernatants were collected 24–48 hours post-infection. Further cell treatments are depicted below in appropriate protocols. Virus particles were pelleted from the supernatants through 25% glycerol-Tris-NaCl-EDTA cushion at 13 K rpm at 4°C and re-suspended in appropriate buffers. Infections were generally done at a multiplicity of infection of 2. When viral titers were not known (as for rSeV-HA-F_5A_mvpt/Fgfpt, see below), the infectious dose was adjusted so that cellular extracts of compared infections contained similar amounts of viral proteins.

### Recombinant virus rescue and virus stock production

**Figure 1:**
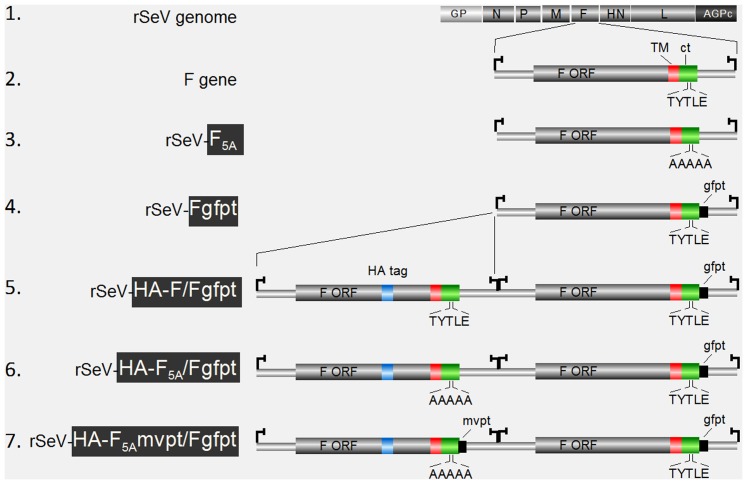
Schematic representation of the recombinant SeV genomes. **1**. Genes encoding N, P, M, F, HN and L are flanked by genomic promoter (GP) and complement of anti-genomic promoter (AGPc). **2**. Blow up of the F transcription unit as present in wt SeV genome, flanked by the start/stop transcription signals(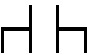). F ORF ecto-domain (in grey), trans-membrane (TM in red) and cytoplasmic (ct in green) domains are outlined, with the TYTLE motif. **3**. Outline of the F ORF as present in rSeV-F_5A_ where the TYTLE motif is replaced by 5 alanines (5A). **4**. Outline of the F gene as present in rSeV-Fgfpt, with insertion in the 5′ UTR of a sequence derived from the green fluorescent protein as target of a specific siRNA (gfpt). **5**. Outline of rSeV-HA-F/Fgfpt carrying an additional F gene inserted between the M and F genes. The additional wt-F ORF harbours an HA tag inserted at position aa 405 (HA tag in blue, see also figure S1). The endogenous F gene harbours the gfpt sequence. **6**. rSeV-HA-F_5A_/Fgfpt. As in 5., except that the TYTLE motif of the additional HA-F ORF harbours the TYTLE/5A mutation. **7**. rSeV-HA-F_5A_mvpt/Fgfpt. As in 6., except that a sequence derived from the measles virus P gene (mvpt) has been inserted in the 5′UTR of the additional F gene as target for a specific siRNA. More details about the rSeV's can be found in Materials and Methods and about the F ORF and its modifications in supplemental figure S1. Only highlighted parts (white on black) of the virus denominations were used for the annotation of the viruses in the figures and in the text.

**Figure 2:**
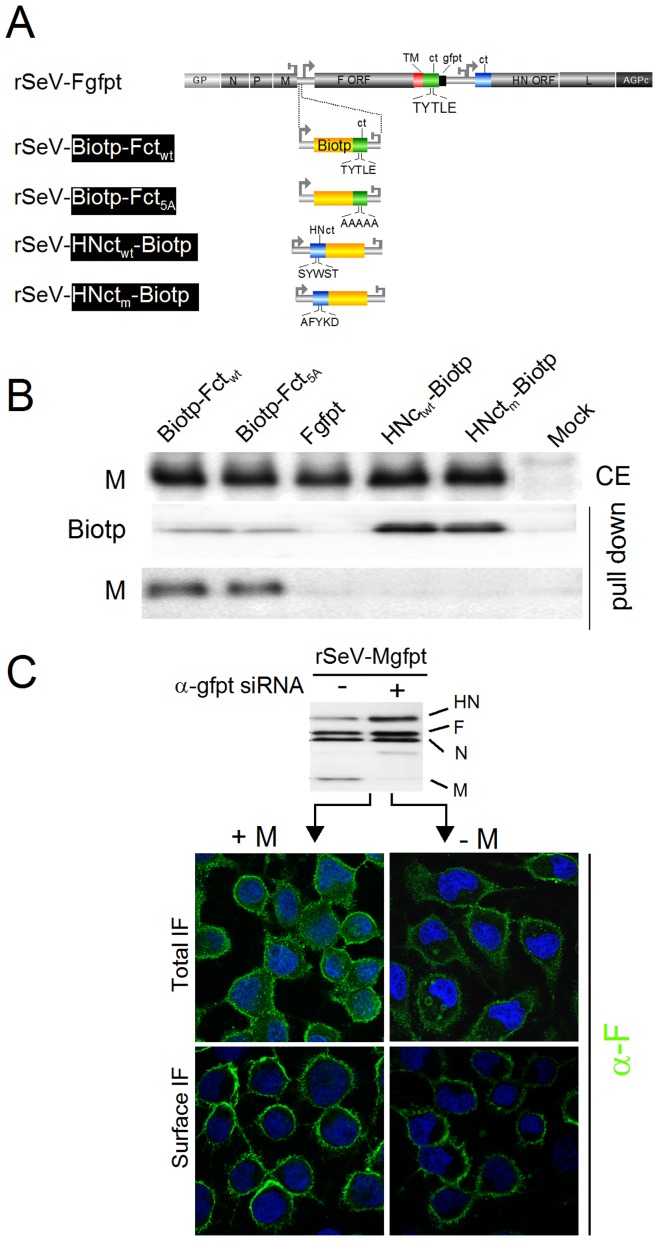
F-M interactions and movement dependency. **A**. Outline of recombinant SeV's harbouring an additional transcription unit expressing the F cytoplasmic tail (ct, green) in its wt (Fct_wt_) or mutated (Fct_5A_) configuration fused to a peptide containing a biotinylation site (Biotp, yellow). Corresponding constructs, harbouring the HN cytoplasmic tail (ct, blue) are equally shown, carrying the wt sequence (HNct_wt_) or a mutation in the SYWST motif (HNct_m_, [11]) Fgfpt: as in figure 1. Note that the endogenous wt-F is open to conditional suppression by α-gfpt siRNA. **B**. α-gfpt siRNA expressing MDCK were infected with the indicated viruses in medium supplemented with 1 mg/ml of biotin. Thirty hours post infection cellular extracts were prepared and incubated with streptavidin agarose resins to pull down Fct and HNct fused to Biotp. Upper panel: Western blot analysis recording cellular levels of M (CE). Lower panels: Western blot analysis of the pull down samples probed with α-M, α-Fct or α-HNct (pull down). **C**. MDCK cells expressing or not α-gfpt siRNA (+/−) were infected with rSeV-Mgfpt, a recombinant virus expressing a M protein open to conditional suppression [15]. The infected cells were either grown in Petri dishes for cellular extract preparation or seeded on coverslips for confocal imaging. Upper panel: Western blot analysis of the cellular extracts prepared 36 hours post infection using an antibody cocktail shown in figure 3 to document M protein suppression. Lower panels: Confocal images of HA-F obtained before (Surface IF) or after (Total IF) cell permeabilization with α-F as primary antibody.

Recombinant SeV's were rescued form full length genome cDNAs exactly as described in Mottet et al. [15]. In brief, BSRT7 cells were transfected with the full length genome cDNAs along with pTM1 based plasmids expressing the N, P/Cstop and P viral proteins. Forty-height hours post-transfection, transfected cells were treated with 1.5 µg/ml of acetylated trypsin, collected 24 hours later and injected into 9 day old embryonated chicken eggs. After three days of incubation at 33°C, the allantoic fluid (AF) was collected and presence of virus was checked by PAGE analysis of a virus pellet obtained from 1 ml AF. When required, further egg to egg passages were made. Schematic descriptions of the rSeV's genomes rescued in this work are presented in figures 1 and 2A (see also below) and conditions and results of rescue attempts are summarized in Table 1. Exceptionally, rSeV-HA-F_5A_mvpt/Fgfpt rescue, requiring suppression of the HA-F_5A_ during rescue, was performed in BSRT7 cells constitutively expressing an siRNA directed against a target sequence originated from the measles virus P gene (α-mvpt siRNA, see below as well). In this protocol, 24 hours post transfection, transfected BST7 cells were cultured in MEM without serum supplemented with 2-fold the amount of vitamins and amino acids and with 1.5 µg/µl acetylated trypsin to allow cell to cell virus transmission. For cell to cell passage, cell supernatants containing virus particles were further treated with 5 µg/µl acetylated trypsin (Sigma) 15 min at 37°C to optimize F cleavage. Virus stock titers obtained on LLCMK2 cells according to Sugita et al. [16] are presented in Table 1. Titers of rSeV-HA-F_5A_mvpt/Fgfpt could not be obtained due to lack of LLCMK2 cells expressing α-mvpt siRNA.

**Table 1:** Summary of recombinant virus properties.

rSeV-cDNA	F gene(s) features								Virus obtained	Virus titers
	Additional F				Endogenous F					Pfu/ml
	wt	5A	Suppression	Expression	wt	5A	Suppression	Expression		
rSeV	-	-	-	-	yes	-	-	yes	yes	2.0×10^9^
rSeV-F_5A_	-	-	-	-	-	yes	-	-	no	NA
rSeV-Fgfpt	-	-	-	-	yes	-	yes/gfpt	yes	yes	3.7×10^8^
rSeV-HA-F/Fgfpt	yes	-	-	yes	yes	-	yes/gfpt	yes	yes	1.9×10^7^
rSeV-HA-F_5A_/Fgfpt	-	yes	-	no	yes	-	yes/gfpt	yes	yes*	ND
rSeV-HA-F_5A_mvpt/Fgfpt	-	yes	yes/mvpt	yes	yes	-	yes/gfpt	yes	yes**	NA

- wt: TYTLE motif in wt protein.

- 5A: TYTLE replaced by 5 alanine.

- Suppression: amenable to suppression by appropriate siRNA. mvpt: measles virus P gene target.

gfpt: green fluorescent protein gene target.

- Expression: possible when not suppressed.

* Virus obtained, but HA-F_5A_ not expressed due to a STOP codon in ORF. -

** Virus obtained only when HA-F_5a_ is suppressed. -

- ND: not done.

- NA: not possible.

### Constructions of full length cDNA plasmids

All the cloning procedures to generate the full length cDNA genomes were performed with home-made cloning strategies, including fusion PCR's, using as backbone the F5 cDNA (Fl5) described in Mottet et al. [15]. Fl5 contains an additional extra-cistron between the M-F genes harbouring a unique MluI site allowing insertion of a supplemental gene. rSeV-F_5A_ cDNA was derived from Fl5 by substitution of the sequence encoding TYTLE motif in the cytoplasmic tail of F with 5 alanines (5A, figures 1 and S1). rSeV-Fgfpt cDNA was derived from Fl5 by insertion of a green fluorescent protein sequence target (gfpt) in the 5′ un-translated region (UTR) of the F gene (see [15] and [14] for similar insertion in rSeV-Mgfpt). The F gene harbouring gfpt is open to expression suppression by its cognate siRNA [14], [15]. rSeV-HA-F/Fgfpt cDNA was derived from rSeV-Fgfpt by insertion in the MluI site of a supplementary F gene modified to harbour a sequence encoding a HA-tag (for sequence and position in the gene, figure S1). Possible side effects of the HA tag on F function was verified by assessing the ability of the HA-F to complement for endogenous F suppression in the virus particle production (see figure S2). rSeV-HA-F_5A_/Fgfpt was prepared by inserting as supplementary gene a TYTLE/5A version of the HA-F gene (as here above). Finally, rSeV-HA-F_5A_mvpt/Fgfpt was obtained by introduction, in the 5′ non-coding region of the inserted HA-F_5A_ gene, of a 3-folds repeated specific siRNA target 20 nt long sequence originating from the measles virus P gene (measles virus P target, mvpt, Denis Gerlier et al., manuscript submitted) allowing suppression of HA-F_5A_ in BSRT7 cells expressing α-mvpt siRNA. The rSeV-Biotp-Fct_wt_, rSeV-Biotp-Fct_5A_, rSeV-HNct_wt-_Biotp, rSeV-HNct_m-_Biotp _-_cDNAs (figure 2A) were derived from rSeV-Fgfpt by insertion in the MluI site of the supplementary genes expressing a peptide harbouring a biotinylation site (Biotp) fused to Fct in their wild type (wt)t or mutated configuration. Similar constructs harbouring instead the HNct (rSeV-HNct-Biotp) were prepared. Biotp, a 96 aa polypeptide derived from pcDNA6/BioEase-DEST plasmid (Invitrogen life technologies), was fused to Fct or HNct according to the supplier's protocol by P.O. Vidalain (Pasteur Institute, Paris) using the pcDNA6BioEase Gateway Biotinylation System. All the rSeV cDNA were sequenced for the modified parts and all obey the rule of six [17]

### Antibodies

The following antibody preparations were used in Western blots as primary antibodies: Rab-HN_SDS_, -M_SDS_, -N_SDS_ and -F_SDS_ (described in [15], ), α-Fct, a rabbit serum raised against a 21 aa peptide proximal to the Fct (see figure S1), α-HA, a mouse monoclonal anti-HA tag (clone 16B12, Covance,) and α-actin (clone 4, Roche). α-HA (Covance) was also used in immunoprecipitations. For immunofluorescent assays, the following mouse monoclonal antibodies were used: α-N (clone M6), α-HN (clone 68) and α-F (clone M38) obtained from Allen Portner (St Jude Children's Research Hospital, Memphis, Tennessee), α-M (clone 383) obtained from Claes Orvell (Laboratory of Clinical Virology, Huddinge Hospital, Sweden) as well as a rat monoclonal α-HA (clone 3F10, Roche). α-calnexin (ER marker) and α-GPP130 (Golgi marker) rabbit polyclonal antibodies were gifts from, respectively, Jean Grünberg and Guillaune A. Castillon (University of Geneva, Faculty of Sciences).

### Total cell immunoprecipitations

Infected MDCK cells were lysed in Lysing buffer II (150 mM NaCl, 1% Sodium deoxycholate, 1% Triton X-100, 0.1% SDS and 50 mM Tris-HCl pH 8.3). The clarified cell extracts were incubated with the appropriate antibody for 2 hours at 4°C, before incubation with Protein-A Sepharose for another 2 hour at 4°C. Immune complexes bound to Protein-A Sepharose were washed twice with NET buffer (NaCl 150 mM, EDTA 5 mM, Tris-HCl pH 7.8 50 mM, NP40 2%) and once with Washing buffer (LiCl 500 mM, Tris-HCl pH 7.8 100 mM, β-mercaptoethanol 1%). The samples were resuspended in SDS PAGE buffer.

### Surface protein biotinylation

Twenty-four hours post-infection, cells were rinsed three times with cold PBS and overlaid with 2 ml of the Biotin reagent (EZ-link™ Sulfo-NHS-SS-Biotin, Thermo-Scientific) diluted in PBS at 0.4 mM. After 30 minutes of incubation at 4°C, cells were rinsed five times with PBS and lysed in lysing buffer I. Biotinylated surface proteins were recovered using streptavidin agarose as described for streptavidine agarose pull-down assays (see below).

### Western blot analysis

Western blot methodology has been previously described [15], [20]. Analysed samples were resuspended in SDS PAGE sample buffer and electrophoresed on 17.5% polyacrylamide gel. Gel transfer was made onto polyvinylidene difluoride membrane (PVDF, Millipore) using Trans-Blot SD Transfer Cell (Biorad). Appropriate primary antibodies and corresponding anti-mouse or anti-rabbit horseradish peroxidase (HRP)-coupled secondary antibodies (Biorad) were then applied (see figures). Proteins were detected using the PNR 2106 ECL Western Blotting Detection System (Amersham). Blot imaging was done using a Fujifilm LAS-4000. In every experiment, the same numbers of cells were plated and infected. The same fractions of cellular extracts and concentrated cell supernatants (virus particles) were analysed.

### Isotopic radio-labelling of cells

For prolonged ^35^S-methionine/cysteine radiolabeling, 16 hours post infection, infected cells were incubated cells with FCS free medium containing 1/10^th^ the amount of methionine and cysteine and 30αCi/ml of ^35^S-methionine and ^35^S-cysteine (Pro-mix-[^35^S]-Amersham, Biosciences). For pulse chase radio-labelling, infected cells were incubated with serum free medium deprived of methionine and cysteine (Sigma) for 30 minutes and then pulse labelled for 10 minutes with 300 µCi of ^35^S-Pro-Mix. At the end of the pulse, cells were either immediately collected or chased for 1 hour or 2 hours in medium supplemented with 10 mM of cold methionine and cysteine. When directly analysed by PAGE, the dried gel was then processed for radioactive protein band detection using a Typhoon FLA 7000 Phospho-Imager (GE Healthcare).

### Endo-H treatment assays

After 0, 1 or 2 hours of pulse-chase radiolabelling, cellular extracts were collected and F protein was immune-precipitated (Total IP, see above) using an anti-HA. After the Washing buffer lavage, the immune-precipitates were processed for endoglycosidase-H treatment using 100 Units of Endo-H (P0702S, New England Biolabs) according to the manufacturer protocol. At the end of the incubation period (1 hour, room temperature), the treated samples were re-suspended in SDS PAGE sample buffer, directly analysed by PAGE and processed as above.

### Streptavidine agarose pull-down assays

MDCK cells were infected with the indicated viruses in medium supplemented with 1 mg/ml of biotin (product B4639, Servilab, France). Pull down with streptavidin agarose resins was performed according to the manufacturer's instructions (Pierce Streptavidin Agarose Resins, Thermo-Scientific). Shortly, 30 hours post infection cells were washed three times with PBS and lysed in lysing buffer I (NaCl 10 mM, Tris-HCl pH 8.0 50 mM, NP40 0.6%). One hundred µl of virus-containing cell culture lysates were incubated with 20 µl of streptavidin agarose for 2 hours at 4°C with occasional shaking. After precipitation, streptavidin agarose was pelleted and washed 5 times with NET buffer (NaCl 150 mM, EDTA 5 mM, 50 mM Tris-HCl pH 7.8, NP40 2%). The elution of bound complexes was performed with SDS PAGE sample buffer.

### Immunofluorescent staining and confocal microscopy

MDCK cells were grown on sterilized coverslips coated with poly-lysine (Sigma). Twenty-four hours post infection, cells were rinsed with 20 mM Hepes pH 7.5-buffered DMEM and fixed with 4% paraformaldehyde in H_2_O pH 7.3 (PFA) 15 minutes at room temperature. For surface immunofluorescence staining (Surface IF) primary antibodies were added *in situ* and secondary antibodies on the PFA fixed cells. In the case of total immunofluorescence staining (Total IF), PFA fixed cells were further permeabilized using methanol (−20°C) or 0.1% saponin in Phosphate Buffered Saline lacking Mg^++^ and Ca^++^ (PBS−/−) containing 1% BSA. Staining of infected cells was performed using primary antibodies described above. Secondary antibodies used were TRITC-conjugated donkey anti-rat IgG (Jackson) and Alexa488 conjugated goat anti-mouse IgG (Invitrogen). The nucleus was stained with DAPI (Boehringer Mannheim GMBH). Finally, stained cells were mounted in Fluoromount-G (SouthernBiotech). Confocal image analysis was performed with the confocal microscope LSM700 (Carl Zeiss) via 63×/1.4 oil immersion objective. Zen software was used for acquisition and then for analysis and treatment of confocal images.

## Results

### Alanine substitution of the F cytoplasmic tail TYTLE motif is detrimental for virus rescue

**Figure 3:**
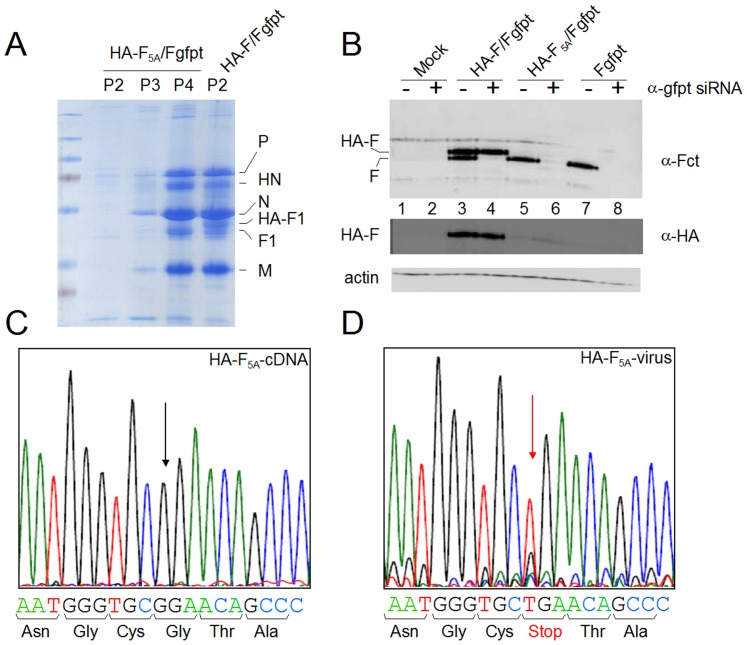
TYTLE/5A mutation of F is detrimental for SeV particle rescue. **A**. Coomassie blue stained PAGE analysis of virus purified from egg allantoic fluids. Lanes P2, P3, P4 (HA-F_5A_/Fgfpt), 2^nd^ to 4^th^ egg to egg serial passages of rSeV-HA-F_5A_/Fgfpt rescue. Lane P2 (HA-F/Fgfpt), 2^nd^ passage of rSeV-HA-F/Fgfpt rescue. **B**. Western blot analysis of MDCK cellular extracts obtained 24 hours after mock infection (Mock), infections with rSeV-HA-F/Fgfpt (HA-F/Fgfpt) or rSeV-HA-F_5A_/Fgfpt (HA-F_5A_/Fgfpt) in conditions of suppression or not of the endogenous wt-F proteins (+/− α-gfpt siRNA). α-Fct : anti Fct antibody. α-HA: anti HA tag antibody. **C**. The cDNA fragment containing the HA-F_5A_ gene was purified after MluI digestion of the rSeV-HA-F_5A_/Fgfpt full length cDNA clone and fully sequenced. **D**. The viral genomic RNA present in rSeV-HA-F_5A_/Fgfpt infected cells was reverse transcribed using a primer of positive polarity in the M gene. The RT product was amplified using a primer of negative polarity in HA tag sequence. The RT-PCR product was then purified and fully sequenced. Arrows in **C**. and **D**. indicate the G to T substitution position leading to introduction of a STOP at position 37 in the F ORF (see also figure S1).

Published data led to the conclusion that the TYTLE motif present in the middle of the F cytoplasmic tail (ct) (figure S1) plays a key role in the formation/production of Sendai virus (SeV) particles (see Introduction). To more precisely describe the mechanism by which this motif intervenes, the rescue of a recombinant Sendai virus harboring alanine substitutions of this motif (rSeV-F_5A_, figure 1 and S1) was attempted. Despite several independent rescue attempts, no virus could be obtained. This negative result was likely due to abolition of F function caused by the TYTLE/5A substitution. To circumvent this problem, we took advantage of our ability to selectively suppress the expression of a viral gene using the siRNA technology. This approach involves insertion, in the gene 5′ un-translated region (UTR), of a short nucleotide sequence originating from the green fluorescent protein (gfpt) and production of cell lines constitutively expressing the α-gfpt siRNA [15]. The SeV genome was then modified by adding i) the gfpt sequence into the 5′UTR of the F gene and ii) a second transcription unit expressing another copy of the F protein tagged with an HA peptide as a tool to distinguish the two F copies (see Material and Methods and figure S1). This extension of the siRNA selective suppression system, that we call Integrated Suppression Complementation System (ISCS), opens now the possibility to suppress expression of the endogenous F protein in order to evaluate the function (i.e. complementation ability) of the supplemental protein F. The rationale of using this approach was the possibility to rescue a virus based on endogenous wild type F (wt-F) expression, and then to characterize HA-F_5A_ after suppression of wt-F. Two recombinant viruses were then produced expressing the supplemental HA-F in wt or mutated versions called rSeV-HA-F/Ffgfpt and rSeV-HA-F_5A_/Fgfpt (see figures 1 and S1). Virions from the rescue of rSeV-HA-F/Fgfpt were quantitatively recovered after the second serial passage in eggs (figure 3A, HA-F/Fgfpt, P2, Table 1). Wt and HA-tagged copies of F1 protein exhibited slightly different electrophoretic mobility just below the major N protein (in allantoic fluid, the F protein is efficiently cleaved into F1/F2). An upper band is visible in rSeV-HA-F/Fgfpt corresponding to the HA-tagged F1 (HA-F1). Accordingly, upon analysis of infected cells (where F is not cleaved), α-Fct immunoblotting revealed two closely migrating bands, the upper one being identified by its exclusive recognition by α-HA immunoblotting (Figure 3B, lanes 3) reactivity. Furthermore, the lower wt-F band detected by α-Fct antibody selectively disappeared in extract from α-gfpt siRNA expressing cells infected with rSeV-HA-F/Fgfpt (figure 3B, compare lanes 3 and 4). Rescue of rSeV-HA-F_5A_/Fgfpt took four serial passages in eggs (figure 3A, HA-F_5A_/Fgfpt, P2–P4). By comparison with rSeV-HA-F/Fgfpt, rSeV-HA-F_5A_/Fgfpt virions were devoid of the HA-F1 upper band (Figure 3A, HA-F1) and upon infection of MDCK cells, only the lower wt F band sensitive to α-gfpt siRNA silencing could be detected with only trace amount of α-HA reactive HA-F_5A_ band (figure 3B lane 5 and 6). Taking advantage of the HA tag sequence in the supplementary F gene, a RT-PCR product of this gene was amplified and sequenced. In comparison with the sequence of the plasmid cDNA at the origin of the rSeV-HA-F_5A_/Fgfpt (figure 3C), the sequence of this RT-PCR product showed a predominant substitution of a G with a T leading to the introduction of a stop codon in the F ORF at amino acid position 36 (figures 3D and S1). This mutation obliterates the HA-F_5A_ protein synthesis. That the TYTLE/5A mutation was counter-selected in the double F gene virus was in agreement with our inability to rescue a viable rSeV expressing HA-F_5A_ as a unique F copy (Table 1). Together these results supported an essential and required role of the Fct TYTLE motif in the life cycle of SeV, but did not provide any clue about the mechanism involved.

### Rescue of an rSeV expressing an HA-F_5A_ protein

**Figure 4:**
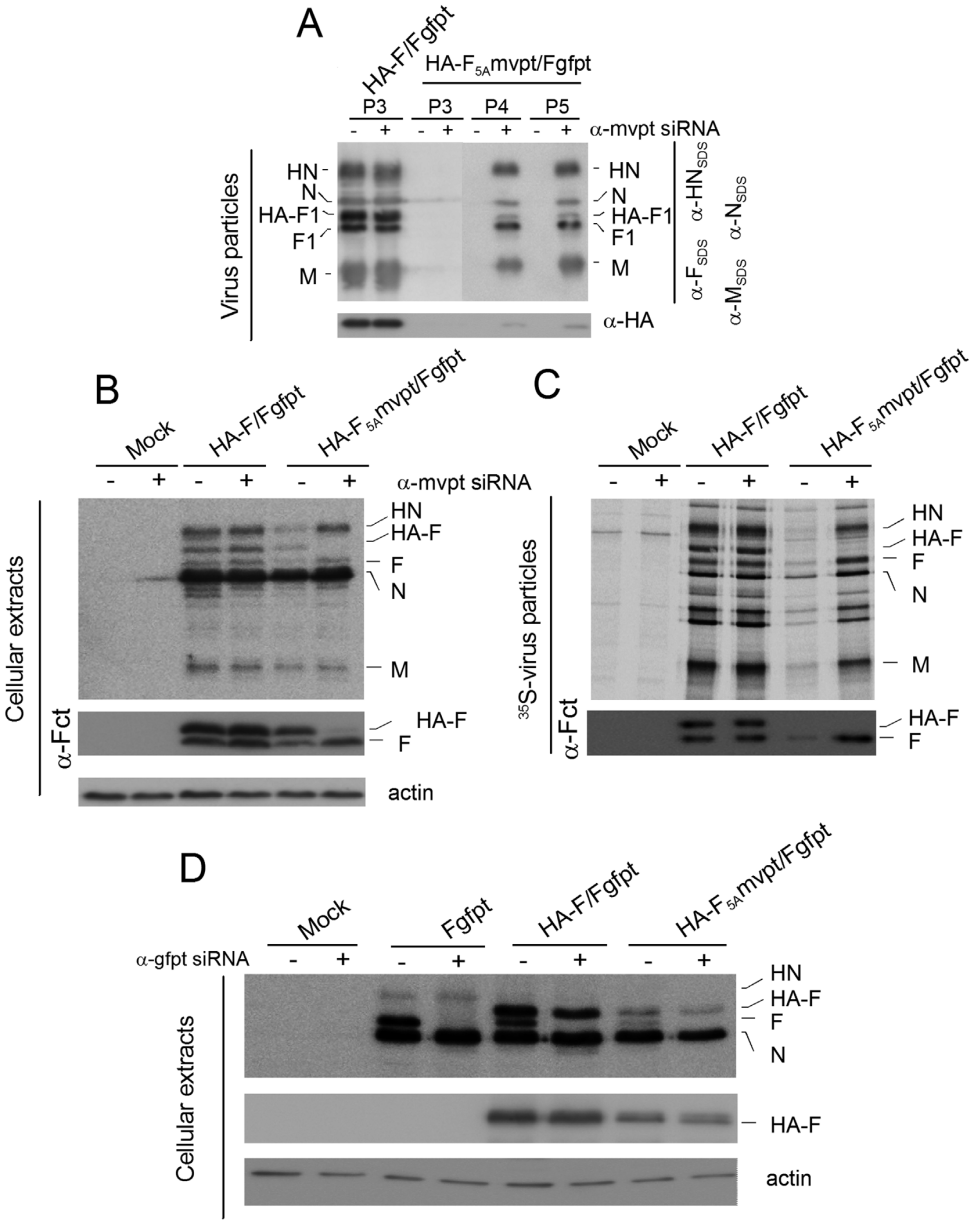
Rescue and analysis of rSeV-HA-F_5A_mvpt/Fgfpt virus. **A**. BSRT7 cells constitutively expressing or not the α-mvpt siRNA (+/−) were used in rescue protocols with rSeV-HA-F/Fgfpt or rSeV-F_5A_mvpt/Fgfpt full length cDNA. Western blot analysis of virus particles purified form these supernatants after 3 passages (P3) for rSeV-HA-F/Fgfpt (HA-F/Fgfpt) or after 3, 4 and 5 passages for rSeV-F_5A_mvpt/Fgfpt (P3,P4,P5, HA-F_5A_mvpt/Fgfpt). α-HN_SDS_, α-F_SDS_, α-N_SDS_, α-M_SDS_: antibody preparations raised against the SDS denatured proteins. α-Fct, α-HA: as in figure 2. **B**. Cellular extracts of BSRT7 cells, constitutively expressing or not the α-mvpt siRNA (+/−) and infected with rSeV-HA-F/Fgfpt or rSeV-F_5A_mvpt/Fgfpt virus preparations obtained as shown in (**A**), were analysed by Western blots using the indicated antibody preparations. Actin: used as loading control. **C**. BSRT7 cells infected as shown in (**B**) were radio-labelled with ^35^S methionine and cysteine. Purified virus produced in the supernatants were directly analysed by PAGE and detected by autoradiography (upper panel) or detected by Western blots using α-Fct (lower panel). **D**. Cellular extracts of MDCK cells constitutively expressing or not α-gfpt siRNA (+/−) and infected with the virus preparations described in (**B**) plus the rSeV-Fgpt virus were analysed by Western blots using the mixed antibody preparations indicated in (**B**, upper panel) for the upper panel, α-HA for the middle panel and α-actin for the lower panel.

To express HA-F_5a_ in the context of SeV infection, a further refinement of the ISCS approach was developed. This involves the additional possibility of alternatively suppressing the supplemental HA-F or the endogenous wt-F, an approach called biG-biS (for two genes-two suppressions), technically developed by one of us (Denis Gerlier, manuscript submitted). To achieve this goal, another siRNA target sequence, originating from the measles virus P gene (mvpt), was inserted in the 5′ UTR region of the supplemental HA-F_5A_ gene, creating rSeV-HA-F_5A_mvpt/Fgfpt (figure 1). rSeV-HA-F_5A_mvpt/Fgfpt opened the possibility to alternatively suppress HA-F_5A_ gene expression in the context of α-mvpt siRNA or wt-F in cells expressing α-gfpt siRNA. In parallel, a BSRT7 cell line was derived constitutively expressing the α-mvpt siRNA. Using this cell line, with a regular BSRT7 cell line as control, rSeV-HA-F/Fgfpt and rSeV-HA-F_5A_mvpt/Fgfpt viruses were successfully rescued and amplified without any passage into fertilized eggs. rSeV-HA-F/Fgfpt virus particles were recovered after three serial infections in both regular BSRT7 or BSRT7 expressing α-mvpt siRNA (figure 4A, HA-F/Fgfpt, P3, −/+ α-mvpt siRNA lanes). While no virus could be recovered in BSRT7 cells allowing the expression of HA-F_5A_, rSeV-HA-F_5A_mvpt/Fgfpt virus was recovered and amplified into α-mvpt siRNA expressing cells at passage 4 (figure 4A, +α-mvpt siRNA lanes, P4). Because of the efficient α-mvpt siRNA-mediated suppression, HA-F_5A_ was barely detected in these virus particles compared to wt HA-F (figure 4A, P4 and P5). Figure 3B presents the analysis of the viral protein expression in −/+ α-mvpt siRNA expressing BSRT7 cells infected with the double F viruses. As expected, α-mvpt siRNA expression has no consequence on the viral protein pattern in rSeV-HA-F/Fgfpt infected cells (figure 4B, HA-F/Fgfpt, − or + α-mvpt siRNA lanes). In rSeV-HA-F_5A_mvpt/Fgfpt infected cells, however, in conditions of HA-F_5A_ expression (− α-mvpt siRNA lane) a slight deficit in HN and F relative to N (or M) is visible in comparison with the condition of HA-F_5A_ conditions. This deficit appears no longer present upon HA-F_5A_ suppression (+ α-mvpt siRNA lane). Expectedly, viral particle production in figure 4B, detected this time by direct analysis of radiolabelled virus, was severely compromised only in conditions of HA-F_5A_ expression (figure 4C, -α-mvpt siRNA lane). In the end, these results definitely demonstrate the detrimental role of HA-F_5A_ in the production of virus particle. In addition, the successful rescue of rSeV-HA-F_5A_mvpt/Fgfpt opened the possibility to determine the mechanism by which HA-F_5A_ blocks virus recovery since, by infection of cells expressing a α-gfpt siRNA, the HA-F_5A_ effect can be studied in the absence of the endogenous wt-F. Indeed, suppression of the endogenous F is effective regardless of the virus (Fgfpt, HA-F/Fgfpt, HA-F_5A_/Fgfpt, + α-gfpt siRNA lanes) without affecting the expression of the supplemental HA-F, be it wt or mutated (figure 4D).

### Biochemical analysis of HA-F_5A_

**Figure 5:**
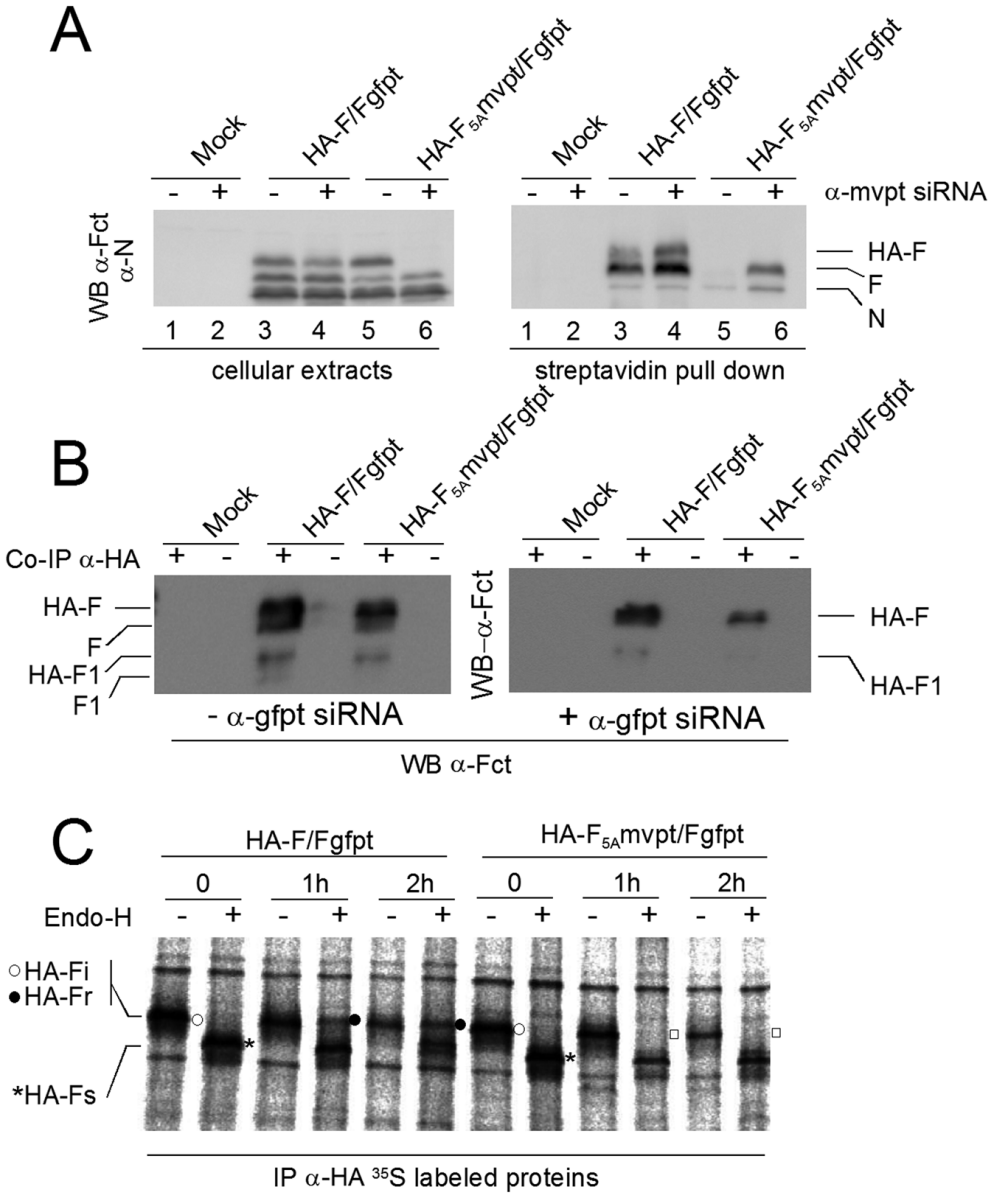
Biochemical characterization of the HA-F_5A_ protein. **A**. BSRT7 cells expressing or not α-mvpt siRNA (+/−) were infected with the indicated viruses. At 24 hours post infection, cells were biotinylated *in situ* and a fraction of cellular extracts was analysed directly by Western blotting (left panel). The remaining was submitted to streptavidine bead pull down before analysis (right panel). Western blots were probed with α-Fct and α-N. **B**. Infections of MDCK cells, expressing or not α-gfpt siRNA. Forty hours post infection total cell immunoprecipitations were performed using or not α-HA antibodies (+/−). Immune-precipitates were analysed by Western blot using α-Fct. Cellular inputs of this experiment can be seen in figure S5. **C**. Infections of MDCK cells expressing α-gfpt siRNA were performed with the indicated viruses. Sixteen hours post infection, the infected cells were pulse-chase radiolabelled. HA-F proteins were immunoprecipitated using α-HA and then treated or with Endo-H (Endo-H, +/−). Digested samples were analysed by PAGE and detected by autoradiography. HA-Fi (○): initial HA-F recovered at the end of the pulse and not treated with Endo-H. HA-Fs (*): sensitive to Endo-H digestion. HA-Fr (•): resistant to Endo-H and (□): shows lack of resistant HA-F_5A_.

Surface expression of the F proteins was tested after biotinylation of cells *in situ*. BSR-T7 cells were used expressing or not α-mvpt siRNA, creating conditions where HA-F_5A_ is respectively suppressed or not. In this way, HA-F_5A_ surface expression and its possible impact on wt-F expression could be investigated. Figure 5A (left panel) shows effective HA-F_5A_ suppression (compare lane 5 and 6, cellular extracts). Note that HA-Fwt is, expectedly, not affected by α-mvpt siRNA expression. When surface biotinylated proteins were pulled down by streptatvidin (right panel), both HA-F and F were recovered when HA-F is in its wt configuration (regardless of α-mvpt siRNA expression, lanes 3 and 4). This contrasts with the absence of recovery of both HA-F_5A_ and wt-F when HA-F_5A_ is not suppressed (− α-mvpt siRNA, lane 5), a wt-F that is normally recovered in the pull down when HA-F_5A_ is suppressed (+ α-mvpt siRNA, lane 6). It turns out, that not only HA-F_5A_ is not expressed at the cell surface, but, moreover, that wt-F is not detected at the cell surface when co-expressed with HA-F_5A_.

Normally, F proteins are expressed at the cell surface and inserted in the viral envelope as a trimer. To investigate whether wt-F and HA-F's could form mixed oligomers, recovery of HA-F were done using α-HA immunoprecipitation. Western blots probed with α-Fct, scoring both F and HA-F show that the two proteins migrate as diffuse F and F1 doublets in the gel (figure 5B, left panel). These diffuse bands were, however, resolved into unique sharp bands when the endogenous F was suppressed (figure 4B, right panel), indicating that wt-F could co-precipitate with HA-F, as well as HA-F_5A_.

Glycoproteins are modified in the ER lumen by addition of a high mannose glycan moiety. This is sequentially trimmed by mannosidases and when the glycoprotein reaches the Golgi apparatus, new complex sugars are added. Endoglycosidase H (Endo-H) removes the high mannose glycan moiety as it stands in the ER, but not after the glycoprotein has reached the Golgi. Resistance to Endo-H cleavage is then used to test the progression of the protein from the ER to the Golgi in transit to the cell surface. Figure 5C documents the Endo-H resistance property of ^35^S-labeling pulse chased F proteins. At the end of the pulse (time 0), both HA-F and HA-F_5A_ showed as expected sensitivity to Endo-H as evidenced by the complete shift of the protein band from a slower migrating glycosylated form (Endo-H −, HA-Fi, ○) to a faster migrating de-glycosylated one (Endo-H +, HA-Fs, *). After 1 hour of chase, while in the case of HA-F, total resistance (time 1 h, Endo-H +, HA-Fr, •) was acquired, this was not the case for HA-F_5A_ (□), a situation that did not change after 2 hours of chase. Thus, the data from Endo-H treatment support a cytoplasmic retention of HA-F_5A_ at the level of the ER. These results demonstrate that HA-F_5A_ remains in the cytosol, likely in the ER compartment and prevents wt-F to reach the cell surface. This is likely achieved by a dominant negative effect exerted through the formation of wt-F/HA-F_5A_ hetero-oligomers.

### Bio-imaging analysis of HA-F_5A_

**Figure 6:**
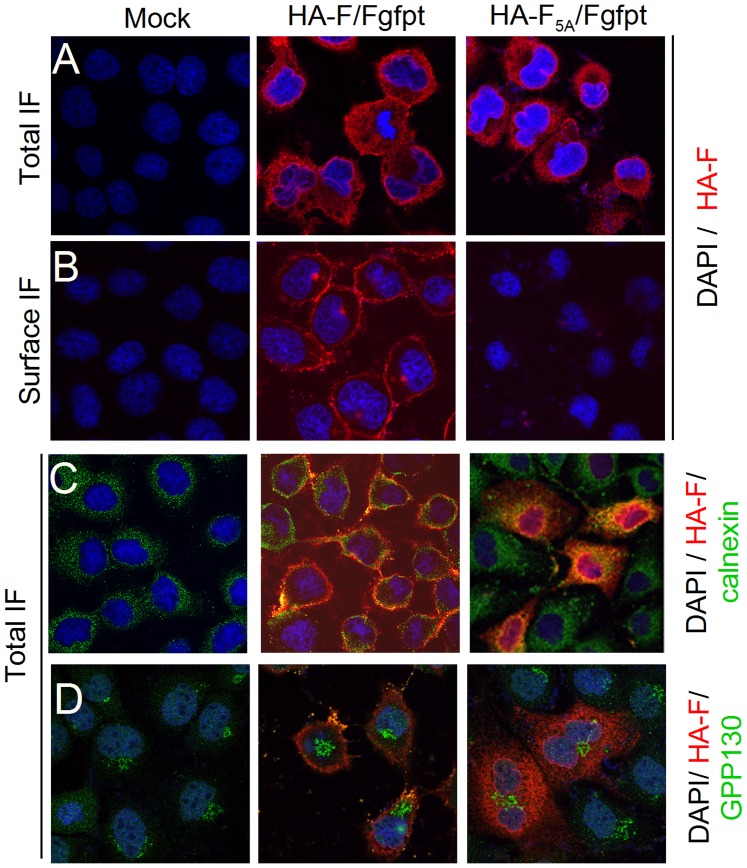
Subcellular localization of the wt-HA-F and HA-F_5A_ in the context of infections. MDCK cells constitutively expressing α-gfpt siRNA were grown on coverslips and infected with rSeV-HA-F/Fgfpt and rSeV-HA-F_5A_mvpt/Fgfpt or kept mock infected. Twenty-four hours post infection, cells were submitted to (**A**.) total immunofluorescence (Total IF) or (**B**.) surface immunofluorescence (Surface IF) protocols. α-HA : primary antibody for both protocols. **C**. and **D**. as in (**A**.), except, α-calnexin (**C**.) or α-GPP130 (**D**.) primary antibodies were added with α-HA. DAPI: nuclei staining. Merged channel images are presented for convenience. Separate channel images for C are presented in figure S4.

**Figure 7:**
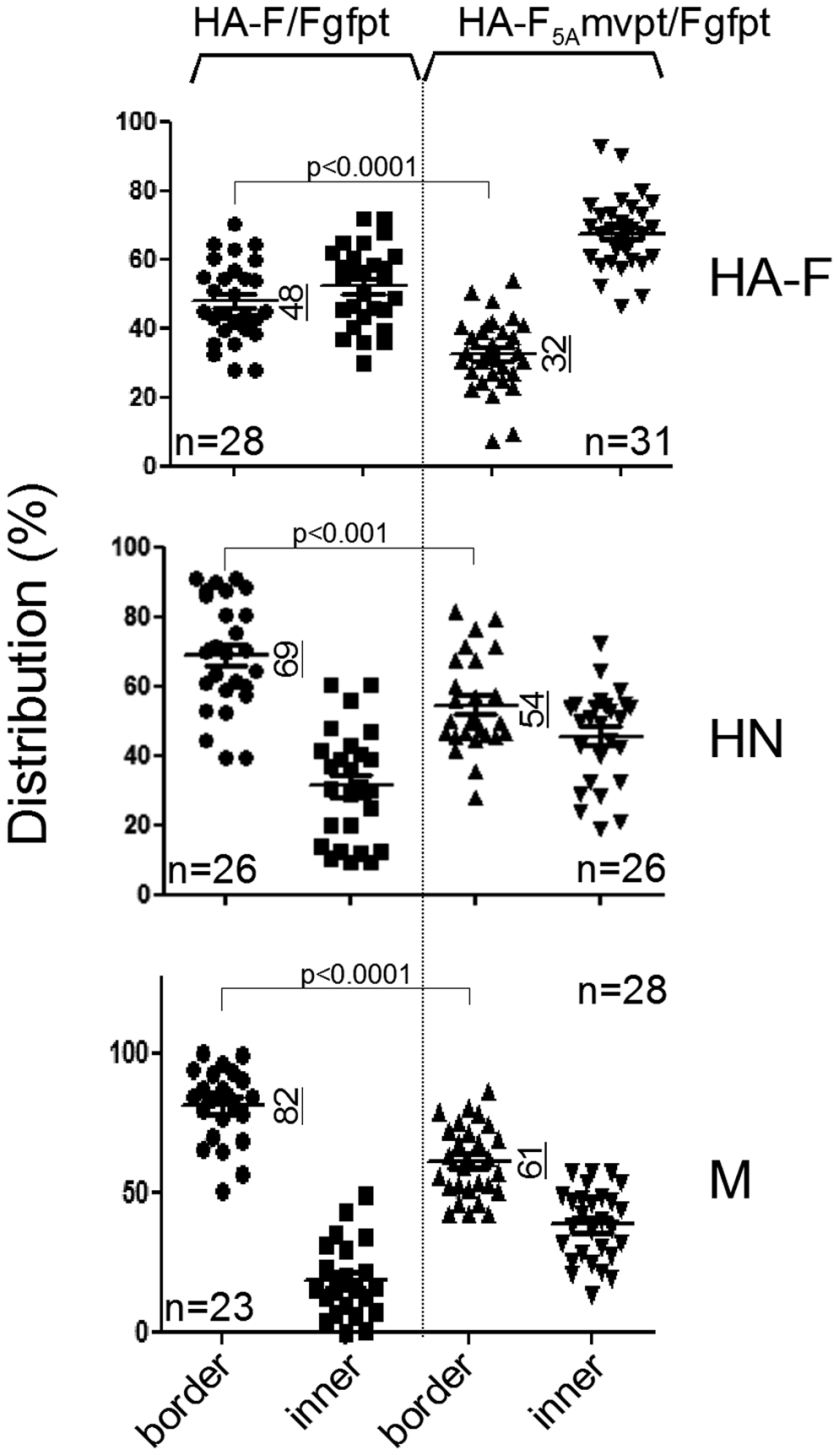
Statistical analysis of F, HN and M distribution in infected cells. Total immunofluorescence confocal images of rSeV-HA-F/Fgfpt (HA-F/Fgfpt) and rSeV-HA-F_5A_mvpt/Fgfpt (HA-F_5A_/Fgfpt) infected α-gfpt siRNA expressing MDCK cells as those presented in figures 6 and 8 were analysed through a Metamorph program presented in supplemental figure S3, designed to quantify the fractions of the proteins HA-F, HN and M at the cell border or in the inner compartment. n = x: refers to the number of cells involved. Graphical representation of the data was executed by Graph Pad Prism 6 software which also provided the statistical calculation of the unpaired two-tailed Student t test. Underlined vertical numbers = numerical averages.

To further document the subcellular localization of HA-F_5A_ and other virus components, confocal microscopy imaging of MDCK infected cells were performed. MDCK expressing α-gfpt siRNA were used to follow the features of the supplemental HA-F protein only, i.e. in conditions of endogenous wt-F suppression. Moreover, these observations were made in two different conditions involving or not cell permeabilization before primary antibody application, leading, respectively, to total cell (Total IF) or only cell surface (Surface IF) immunostaining. Permeabilized HA-F/Fgfpt infected cells stained with α-HA antibodies produced images of HA-F (Total IF) where the protein is seen diffuse in the cytosol with nevertheless visible sharp limits at the outer border that should correspond to cell surface (figure 6A, HA-F/Fgfpt, Total IF). Indeed the cell surface immunostaining shows a ring of F staining (figure 6B, Surface IF). For HA-F_5A_, the pattern is drastically different in that no cell surface staining is visible, in agreement with the failure to recover any biotinylated HA-F_5A_ from the cell surface (figure 5A). Accordingly, the cytosolic staining of HA-F_5A_ (figure 6A, Total IF) shows little evidence of cell border staining. To get statistical values on these staining patterns, a Metamorph program was developed that quantify the inner and border staining of the cells (see figure S3). Figure 7, HA-F panel, presents the results of such quantification. Although the program will still score some HA-F_5A_ staining at the cell border, due to the difficulty of assessing with enough precision the cell limits (cell inner surface is also included in the border zone), it nevertheless provides statistical evidence for changes in staining distribution.

The HA-F_5A_ retention in the ER suggested by the Endo-H resistance test (figure 4D) was further substantiated by performing subcellular localization staining using calnexin and GPP130 as resident ER and Golgi markers, respectively. In figure 6C, calnexin staining appears punctiform and general over the cytosol. Wt-HA-F staining expands most of the time at the cell border past calnexin staining in agreement with its predominant distribution at the cell surface. This is not the same for HA-F_5A_ that largely overlaps with calnexin, as shown better in figure S4, where the individual color channels are shown. Regarding GPP130 staining (figure 6D), it is pretty much clear that there is no overlapping with either HA-F. These images then are compatible with a wt-HA-F transported rapidly (v_1/2_ = 5′, [21]) to the plasma membrane, with no visible accumulation in the cytosolic compartments. In contrast, HA-F_5A_ appears as accumulating in the ER, never reaching the Golgi compartment and the cell surface.

**Figure 8:**
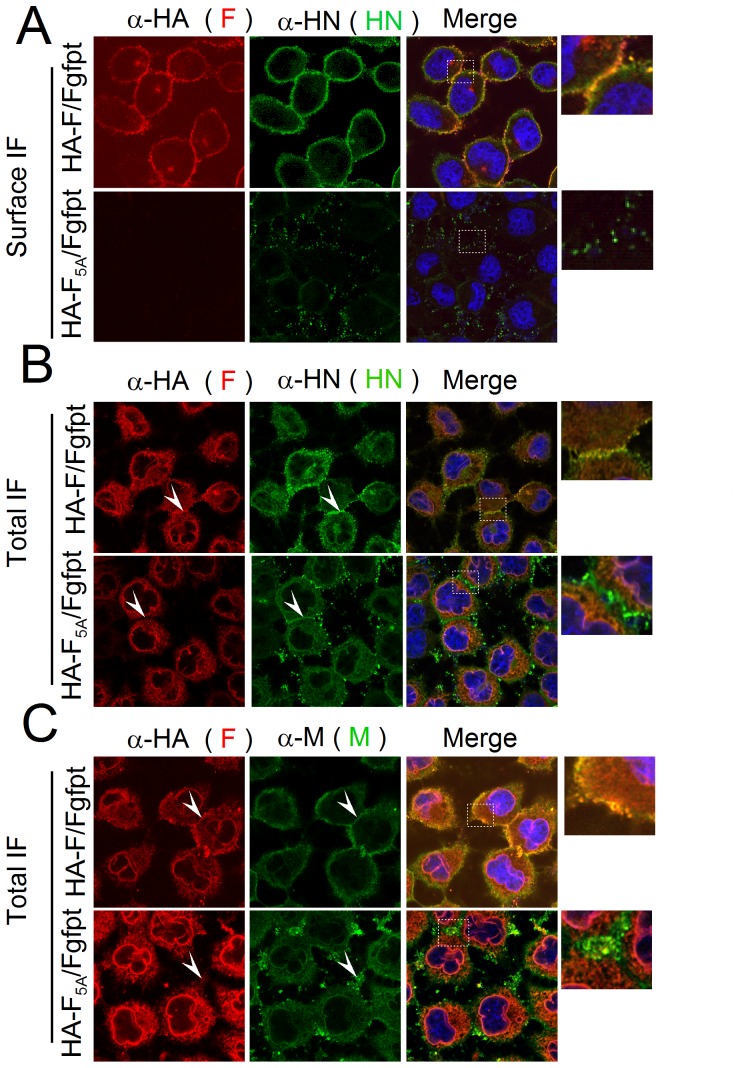
Subcellular localization of the HN and M proteins in the context of wt-HA-F or HA-F_5A_. As in figure 6A, but with the use of primary antibodies α-HN in (**A**) and (**B**) or α-M in (**C**) added with α-HA in all the panels.

Further to defining the changes in F subcellular localization induced by the TYTLE/5A mutations, possible effects of the HA-F_5A_ transport restriction on the two other envelope proteins HN and M were studied. After cell surface immunostaining, wt-HA-F and HN proteins are found to co-localize at the plasma membrane (figure 8A, HA-F/Fgfpt). They both form continuous punctuated rings that partially overlap, although individual spots are visible (see enlarged inlet). Lack of HA-F_5A_ cell surface expression, on the other hand, has a drastic effect, quantitatively and qualitatively, on HN surface expression (figure 8A, HA-F_5A_/Fgfpt). The ring organization is lost and only weak spots are visible (see enlarged inlet). After cell permeabilization, HN is found diffuse in the cytosol and in spotty pattern at the cell to cell junction where there is no overlapping with HA-F_5A_ staining (figure 8B, HA-F_5A_/Fgfpt, see enlarged inlet and white arrow heads). This contrasts with the HN/F co-localization observed at the cell to cell junction in HA-F/Fgfpt infected cells (figure 8B, HA-F/Fgfpt, see enlarged inlet, and white arrow heads). The statistical analysis of the HN distribution presented in Figure 7 (HN panel) recapitulates the trend that sees HN less present at the cell border. Disruption of the ring pattern could not, however, be scored by these measurements.

The M protein can only be observed after cell permeabilization. In the context of HA-F/Fgfpt infection, it is seen diffuse in the cytosol, but also at the cell border reproducing the ring pattern which presumably represents the M inner layer under the plasma membrane. At the cell border, M partially overlaps with wt HA-F (figure 8C, HA-F/Fgfpt, see enlarged inlet, and white arrow heads). In the context of HA-F_5A_/Fgfpt infection, however, M distribution has lost its ring shape at the border and appears more diffuse in the cytoplasm. At the cell to cell junction, it may form randomly distributed patches which rarely overlap with HA-F_5A_ (figure 8C, HA-F_5A_/Fgfpt, see enlarged inlet, and white arrow heads). The general distribution of M in the presence of HA-F_5A_ could be monitored statistically as shown in figure 7, panel M. In conclusion, the bio-imaging analysis of the viral protein distribution in the context of HA-F_5A_, definitively confirmed the lack of cell surface expression of HA-F_5A_, which appears restricted to the ER. Importantly, this restriction of HA-F_5A_ has profound consequences for the two other viral envelope proteins. Both HN and M see their distribution at the cell surface decreased and disorganized compared to the pattern prevailing in the context of wild type F. It therefore appeared that restriction of F_5A_ in the cytosol prevents the normal positioning of HN and M at the plasma membrane.

### Analysis of F-M interactions

In the context of the infection, about 50% of the M protein is found associated with membranes as measured by floatation gradients [20]. Since about the same fraction of M expressed alone from plasmid can bud into vesicles [22], [23], it is assumed that M reaches the site of budding at the plasma membrane on its own. The data presented here in figures 8 and 7 suggest that a fraction of M is transported to the plasma membrane in association with F, since restriction of HA-F_5A_ in the cytosol appears to retain M in the cytosol. To verify this assumption, a method was developed to estimate the interaction of M with the F cytoplasmic tail (ct). This approach was developed because in our hands co-immunoprecipitations of M with the viral glycoproteins was found unreliable, due in part to M sticking non-specifically to protein A sepharose beads. Two rSeV were then generated expressing wt TYTLE or TYTLE/5A Fct's along with two rSeV's expressing the HNct's used here as specificity controls (figure 2A). Pull down assays (figure 2B) demonstrated that M protein was similarly recovered interacting with Fct of wt TYTLE or TYTLE/5A configuration. In contrast, the HNct's and the Fgfpt constructs did not pull down M, showing not only that Biotp was not responsible for the pull down, but also that Fct contains an element of specificity in its interaction with M. Noteworthy, probing the pull down material with antibodies directed against the other viral proteins gave no positive signals (not shown). These results suggest that HA-F_5A_ can still interact with M, and is consistent with a decreased localization of M at the plasma membrane when HA-F_5A_ is restricted to the cytosol.

The next question was whether F-M interaction is required for F transport to the plasma membrane. Using rSeV-Mgfpt [15], subcellular localization of wt-F was analyzed by confocal imaging after conditional suppression of M (−/+ α-gfpt siRNA). Upon M suppression (as demonstrated by Western blotting), wt-F immunostaining at the cell surface was not altered in comparison with that obtained in the presence of M (figure 2C surface IF, compare right and left panels). Thus, the F protein can travel to the plasma membrane independently of M. In contrast, M (or a fraction of it) only reaches plasma membrane in association with F.

## Discussion

**Figure 9:**
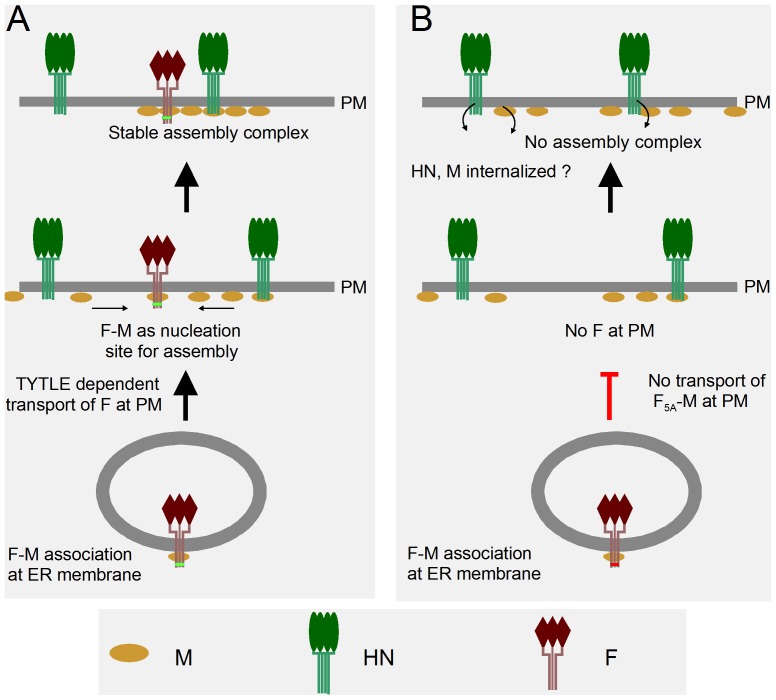
Possible model depicting formation of the viral assembly complex dependent on F. Only the viral membrane proteins F, HN and M are considered for simplicity (see Text for further explanations). **A**. Wild type F interacts with a fraction of M at the level of the ER membranes. This F-M protein complex is transported to the plasma membrane in a TYTLE (small green rectangle) dependent manner. At the cell surface (PM), F-M defines a nucleation site for more M condensation (a fraction of M positioned at PM without F interaction) and for HN recruitment (likely through M). Nucleocapsids (not shown) may now bind to a stable assembly complex made of the 3 membrane viral proteins leading to virus particle budding. **B**. When F is mutated (red rectangle), F-M complex remains in ER due to the absence of the TYTLE motif. Absence of assembly nucleation site at PM results in disorganization of M and HN patterns at PM and stable assembly complex capable of binding nucleocapsids is not formed leading to lack of virus particle production.

When the TYTLE motif present in the cytoplasmic domain of SeV-F was mutated to 5A, no virus could be rescued. When this HA-F_5A_ was co-expressed with wt-F, a virus could be rescued, but the HA-F_5A_ ORF was interrupted resulting in the absence of HA-F_5A_ synthesis. These two results strongly argued for a poisoning effect of HA-F_5A_ affecting viral viability. It is only when virus rescue was done in conditions where HA-F_5A_ was suppressed during rescue that viable virus could be obtained. More than confirming the deleterious effect of HA-F_5A_, the recovery of a virus conditionally expressing HA-F_5A_ provided a tool to study the mechanism by which the mutation affects virus viability, since this recovered double F expressing virus also contained the property of conditionally suppressing wt-F. Then, in conditions of wt-F suppression, we found that HA-F_5A_ remained in the endoplasmic reticulum compartment and failed to reach the plasma membrane. In correlation with this restriction in the cytosol, the pattern of M and HN localization changed in two ways. Their presence at the cell border (for M) or at the cell surface (for HN) was diminished and their distribution was altered, suggesting disorganization relative to the normal pattern. On the basis of these observations and assuming that the TYTLE mutation only comes to arrest the F progression to the cell surface, we propose that, in normal conditions, a F-M complex would form in the cytoplasm (likely at the level of the ER) and migrate to the plasma membrane where it would create a nucleation site at which more M and HN proteins would assemble into a stable complex (figure 9A). In the case where F-M does not reach the plasma membrane, due to restriction of F_5A_ in the ER (a F_5A_ that can still interact with M), this nucleation site would not be set in place preventing the formation of virus particle (figure 9B).

This model is coherent with the failure to rescue a virus expressing only HA-F_5A_ and also the failure to rescue a virus expressing a double F (with HA-F_5A_) since HA-F_5A_ likely acts as a dominant negative protein capable of blocking an HA-F/F hetero-oligomer in the ER. Figures 5A shows, indeed, lack of wt-F at the plasma membrane, when co-expressed with HA-F_5A_ and figure 5B supports interaction of HA-F_5A_ and wt-F. We have not analyzed further the formation of such a trimer in the ER. Interestingly enough, however, by retaining measles virus F in the ER, Plemper and colleagues were able to show that the protein was capable of oligomerization [24], supporting the possibility that SeV F oligomerizes as early as in the ER.

This model recapitulates previous findings about an F-M association already formed in the pre-Golgi compartment [25]. It takes also into account the association of M alone with the membrane [22], [23], a fraction of M that could be recruited to the proposed nucleation site by M-M interactions, and the reinforcement of this binding by the presence of either F and HN [26] to which M is claimed to bind independently [27]. It is not known whether HN possesses in its cytoplasmic tail a plasma membrane addressing motif. HN cytoplasmic tail, however, contains a SYWST motif required for uptake of the protein in virus particles ([11], [12], [14] and manuscript submitted). As mutation of this motif prevents HN uptake in viral particles but not HN cell surface expression, it is likely that HN is recruited in the assembly complex once expressed at the cell surface. This recruitment could be done through HN-M interactions and the reduction of HN cell surface expression (figure 8B) concomitant with the reduction of M level at the cell border (figure 8C) could well reflect the need for HN stabilization by M at the surface, as even in normal conditions a fraction of HN is internalized [14]. However, a direct interaction of HN cytoplasmic tail and M remains to be demonstrated (see figure 2B). Once the patches composed of the viral envelope proteins would be stably organized (and large enough), these could attach the nucleocapsids and form the complete assembly complex ready to bud. When cells infected with either of the two F viruses were immunostained with anti-N antibodies, no difference could be seen in the N pattern, a pattern that was not indicative of an assembly complex to start with (figure S6). On the other hand, membrane isolation by floatation gradients showed that N, likely in the form of nucleocapsid, was attached to membrane fractions even in the absence of any other viral partner (figure S7). This would support a transport of nucleocapsid by itself to the membrane, where interactions with M (or HN, or F?) would bring it to the assembly complex. This model is not exclusive and could as well integrate a beforehand M-nucleocpasid interaction required for recruitment of the nucleocapsids to the membrane as described for measles virus [28]. This M-N complex was moreover shown to reach TritonX-100 resistant membranes (rafts, considered as site of viral assembly), in the absence of H and F [29]. It is difficult here to make a distinction between the faction of viral constituents engaged in the assembly complex leading to viral particle production (for SeV nucleocpasid ∼10%, [30].) and the remaining that accumulates in the cells (in large inclusion bodies, see figure S6).

This model defines F as the key organizer of the assembly complex, a role previously attributed to M, which nevertheless remains important in the process but under the control of F. Notably, suppression of F or M leads to similar decrease in virus particle production [14], an expected result for two players in the same game. This model, however, does not account for the most drastic reduction of virus particle production observed after co-suppression of F and HN (shown in [14]).

In the case of PIV5, F rather than HN was shown dispensable for normal virus budding. By mutations introduced in the cytoplasmic tails of both proteins, however, the extent of the budding defect depended now on the extent of the F cytoplasmic tail truncation supporting here again the importance of the two proteins at a non-defined step [31]. Requirement of the glycoproteins rather than the matrix protein for assembly and budding of enveloped virus-like particles has also been reported in experiments concerning Influenza virus [7].

The identification of TYTLE as involved in transport of F to the plasma membrane opened the possibility to clarify the mechanism of the assembly complex formation. The involvement of tyrosine-based motifs in protein trafficking through ER, to Golgi and plasma membrane has been reported for Alphaherpesvirinae surface glycoproteins [32]. For vesicular stomatitis virus G, this tyrosine-based motif was shown to act in concert with di-acid motifs [33]. For measles virus glycoproteins, such motifs have been shown to influence H and F topology in polarized epithelial cells as well as in lymphocytes [34]. Similarly, a membrane proximal cytoplasmic tyrosine targets HIV GP41 to baso-lateral membranes in polarized MDCK cells [35]. Based on these reports, however, it is difficult to relate TYTLE to a particular generic motif, since they all differ in sequence and amino acid number: YXXL, YXXΦ tyrosine plus di-acids motif D and E (which are both flanking TYTLE). Besides, no effort has been invested from our part in dissecting TYTLE to precisely identify the residue(s) involved. In plasmid based expression of F and estimation of F release in the cell supernatant, substitutions in TYTLE creating TATAE resulted in a significant decrease in F release, although F was found at the cell surface [36]. In the same study, removing TYTLE with deletion of the 24 aa proximal to the C-ter resulted in no F release. This contrasted with the results obtained by us previously in which truncation of 28 aa proximal to the C-ter allowed recovery of a virus (rSeV-Fct_14_, see figure S1), the infection of which led to only a 2-fold reduction of VP production [11]. Rather than comparing two tests that are likely not measuring similar properties, it may be more pertinent to try to reconcile this latter result with the present data. Fct_14_, in contrast to HA-F_5A_, was found expressed at the cell surface. Removing 28 aa (and TYTLE), then, is not equivalent to keeping full length cytoplasmic tail with mutated TYTLE. This could mean that Fct_14_ has lost the controlled transport mechanism provided by TYTLE and travels by default to the plasma membrane. This suggests, also, that Fct_14_ could still interact with M (see below), unless HN complements for F deficiency (see above, synergy and redundancy).

Our model postulates that F interacts with M in the ER and transits to the plasma membrane in complex with M. Interaction of M with the two glycoproteins “in transit through the secretory pathway” has been proposed before in experiments where the glycoproteins were blocked in the transit by low temperature or use of monensin [25]. Unfortunately, no direct interaction, neither co-localization of M with HN or F in the same cells was shown. In the present study, pull down experiments demonstrate that Fct-M interaction is not lost after TYTLE mutation and that this interaction takes place with a cytosolic Fct surrogate. More than supporting an interaction that takes place before F cell surface expression, the pull down assays point to an F-M interaction site distinct from TYTLE. If Fct_14_ remains competent for assembly, and assuming that the F interacts directly with M, then the interaction site would sit in the 14 aa proximal to the trans-membrane region.

In conclusion, the use of an innovative experimental approach involving conditional alternate suppression of two F proteins, one of which mutated and poisonous for virus viability, allowed the deciphering of the steps involved in the formation of paramyxovirus particle in a detail never achieved before

While this manuscript was brought to completion, a publication by Stone and Takimoto [37] was issued that dwells on the critical role of the F cytoplasmic tail in Parainfluenza virus assembly. Using different chimeric rSeVs harboring HPIV1 HN, or F, or HN and F carrying the SeV F cytoplasmic tail, they report on the importance of the SeV F cytoplasmic tail for proper positioning of F at the cell plasma membrane, which in turn influences proper HN, M and N association at this location. If our present results come as a confirmation of their conclusions, they possibly clarify the mechanism by which F plays its critical role; namely by showing that a F-M complex could form in the ER and becomes a nucleation site for assembly at the plasma membrane.

## Supporting Information

Figure S1 **Features of the F ORF pertinent for this study.** HA-F is 581 aa long due to HA tag inserted at position aa 405 in between 2 arginine's (upper right). Upper middle: proteolytic cleavage site of F0, creating the F2/F1 subunits. Upper left: a STOP codon is substituted to glycine 36 in HA-F_5A_ after rescue of rSeV-HA-F_5A_/Fgfpt through normal rescue protocol (figure 2). Bottom part: trans-membrane (TM, red) and cytoplasmic tail (ct green) depicted to emphasize the TYTLE motif in wt-F substituted with AAAAA in F_5A_. Fct14: Fct truncation as present in rSeV-Fct14 from Fouillot-Coriou et al. [11] mentioned in Discussion. α-Fct: outline of the peptide used to raise the α-Fct rabbit serum reacting against wt-F and F_5A_.
(TIF)

Figure S2 **Wt-HA-F fully complements F suppression in the production of viral particles.** MDCK cells expressing or not α-gfpt siRNA were infected with the indicated viruses. At 24 hours post infection, cellular extracts were prepared and viral particles were collected from the supernatants and analysed by Western blots using the indicated antibodies. Left panel shows the suppression of F (α-gfpt siRNA+lanes), better seen on α-Fct WB. Right panel shows that, upon suppression of F, HA-F can fully complement for VP production, as no decrease in VP is observed (HA-F/Fgfpt lanes).
(TIF)

Figure S3 **Statistical analysis of the distribution SeV envelope proteins in infected cells.** **A**. MDCK cells constitutively expressing α-gfpt siRNA were grown on coverslips and infected with rSeV-HA-F/Fgfpt. Twenty-four hours post infection, cells were submitted to surface immunofluorescence (Surface IF) protocols, staining wt HA-F and HN. **B**. The stack of visible light images was processed to obtain the best focus for basic delimitation of cell borders. On the resulting images, cell outlines were provided manually and transferred to images of F and HN staining (merge in A). From these outlines, a cell border zone was delimited by dilatation to +/−10 pixels inside and outside. **C**. Defining the cell border (light blue) allowed definition of a cell inner compartment (dark blue). From each compartment, an event (corresponding to the presence of a given protein, F/HN/M), was identified by a signal having a minimal intensity level. **D**. Events were finally counted from both compartments and percentage of the protein distribution was calculated and presented graphically. As in this case one deals with surface immunofluorescence images of wt F and HN, the results serve as validation for the analysis approach as >85% of the staining is scored as expected in the border compartment. Graphical representation of the data was executed by Graph Pad Prism 6. Underlined vertical numbers = numerical averages.
(TIF)

Figure S4 **Subcellular localization of the wt HA-F and HA-F_5A_ in the context of infections.** The merge images of figure 5C are presented to visualize the prominent peri-nuclear staining of HA-F_5A_ where it co-localizes with calnexin. This contrasts with the ring shape staining of HA-F at the border of the cell.
(TIF)

Figure S5 **Inputs of the co-immunoprecipitations shown in** **Figure 5B** **.** Western blot analysis of the MDCK cellular extract samples used to perform the co-IP presented in figure 4B. The samples were probed with α-Fct to visualize both HA-F and F so to appreciate the ratio of the two proteins in each extract. Quantitatively in excess to the HA-F_5A_mvpt/Fgfpt, the HA-F/Fgfpt samples were revealed with a shorter exposure, although all the samples were part of the same PAGE.
(TIF)

Figure S6 **Subcellular localisation of N is not affected by presence or absence of F at the plasma membrane.** MDCK cells constitutively expressing α-gfpt siRNA were grown on coverslips and infected with rSeV-HA-F/Fgfpt and rSeV-HA-F_5A_mvpt/Fgfpt. Twenty-four hours post infection, cells were submitted to total immunofluorescence protocol. α-HA and α-N were applied as primary antibodies to visualize HA-F and N protein, respectively.
(TIF)

Figure S7 **Part of SeV N associated with cellular membrane is in the form of nucleocapsid.** **A**. rSeV-GFP infected MDCK cells, collected at 30 hours post-infection in 300 µl TNE (Tris-HCl pH 7.5 10 mM, NaCl 50 Mm mM, EDTA 1 mM) −10% sucrose, were disrupted by vortexing with 150 µl of glass beads. The cell lysate (300 µl) were mixed with 900 µl of 90% sucrose in TNE and set at the bottom of SW60.1 Beckman centrifuge tubes (50 µl was set aside representing the input). The floatation gradient was completed with 2.5 ml of sucrose 65% and finally 1 ml of 10% sucrose in TNE. After 12 hours of centrifugation at 12°C, 40 K, three 1.5 ml fractions were carefully collected from the top of the tubes, representing the upper (U), middle (M) and lower (L) samples. Fractions of these samples (50 µl) were mixed with 25 µl of 3× concentrated PAGE sample buffer, boiled and analysed by Western blots using a cocktail of μ-HN, F, N, M antibodies, plus α-GFP; GFP serving as the control protein not associated with membrane. Histogram at right: quantification of HN (light grey bar), F (dark grey), M (black), N (red) and GFP (green) protein fractions from 2 independent experiments. **B**. The N, P and L proteins were expressed from plasmids alone (upper western blot N,P,L; blue bars in histogram) or co-expressed with expression from plasmid of the full-length SeV RNA genome (lower western blot N,P,L +; green bars in histogram) in BSRT7 cells. Cell lysates were then analysed by floatation gradients as in (**A**). Histogram: quantification of two independent experiments. **C**. rSeV-GFP infected MDCK cell lysates prepared by disruption in 0.6% NP40, 50 mM Tris-HCl pH 7.0, 10 mM NaCl (lysing buffer I, [38] or Upper fraction obtained from N, P, L expressing BSRT7 cell floatation gradient analysis as presented in (**B**. N,P,L) were loaded onto linear 20–40% CsCl gradients and centrifuged for 2 hours (12°C, SW 41, 36 K, [38]. Nine 1.3 ml fractions were collected and analysed by Western blots using α-N_SDS_ and density ρ of the fractions was measured by a refractometer. The Western blots were quantified and the N distribution in the gradient reported. Pos: Nucleocapsid from rSeV-GFP infection (histogram green bars), Upper (blue bars) represent floatation gradient fractions obtained from N, P, L expressing cells as described in (**B**). Data obtained from 2 independent experiments. This figure shows that N is found associated with membrane fractions in the context of regular infection (**A**) as well as in the absence of the viral membrane protein expression (**B**). This latter N is found in the form of nucleocapsid in proportion corresponding to that found in regular infection (**C**), demonstrating that nucleocapsid can associate with cellular membrane without interactions with the viral membrane proteins.
(TIF)
