# Supplementary figures and images for: Mutation of the TYTLE Motif in the Cytoplasmic Tail of the Sendai Virus Fusion Protein Deeply Affects Viral Assembly and Particle Production
Source: PLoS One. 2013 Dec 10;8(12):e78074. doi: 10.1371/journal.pone.0078074 (PMC3858230; doi:10.1371/journal.pone.0078074)

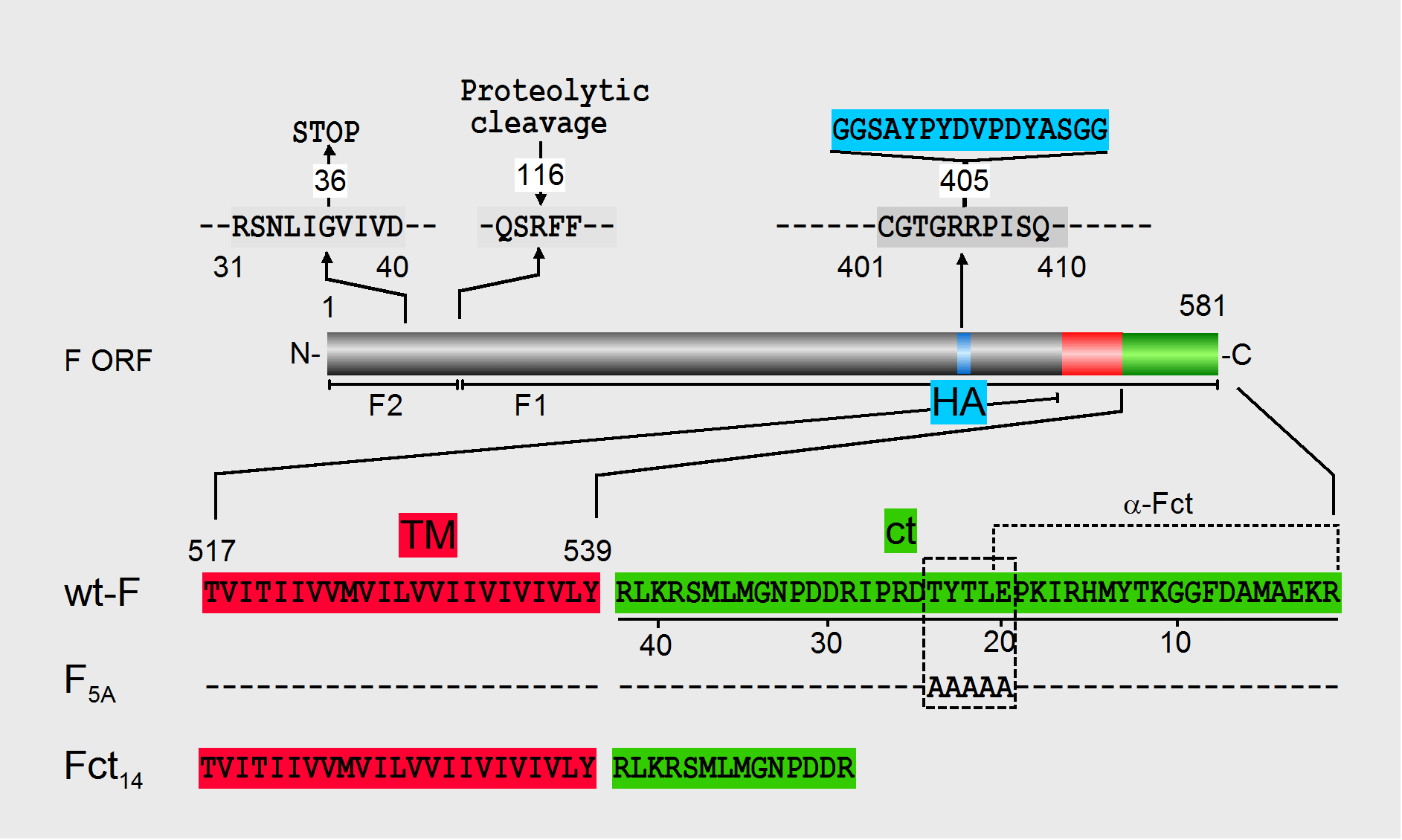

Supplement: Figure S1 — Features of the F ORF pertinent for this study. HA-F is 581 aa long due to HA tag inserted at position aa 405 in between 2 arginine's (upper right). Upper middle: proteolytic cleavage site of F0, creating the F2/F1 subunits. Upper left: a STOP codon is substituted to glycine 36 in HA-F5A after rescue of rSeV-HA-F5A/Fgfpt through normal rescue protocol (figure 2). Bottom part: trans-membrane (TM, red) and cytoplasmic tail (ct green) depicted to emphasize the TYTLE motif in wt-F substituted with AAAAA in F5A. Fct14: Fct truncation as present in rSeV-Fct14 from Fouillot-Coriou et al. [11] mentioned in Discussion. α-Fct: outline of the peptide used to raise the α-Fct rabbit serum reacting against wt-F and F5A. (TIF) [file pone.0078074.s001.tif]

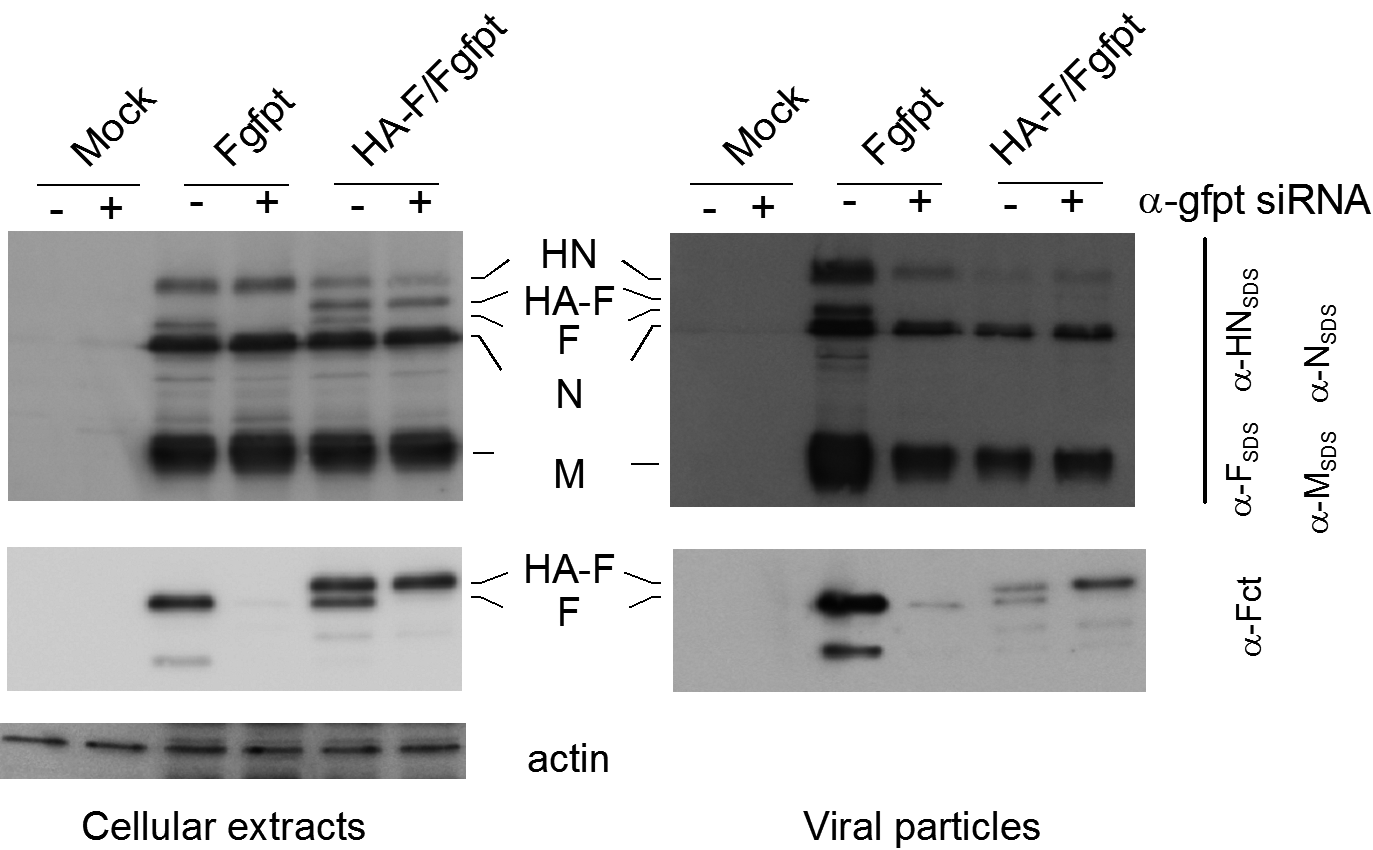

Supplement: Figure S2 — Wt-HA-F fully complements F suppression in the production of viral particles. MDCK cells expressing or not α-gfpt siRNA were infected with the indicated viruses. At 24 hours post infection, cellular extracts were prepared and viral particles were collected from the supernatants and analysed by Western blots using the indicated antibodies. Left panel shows the suppression of F (α-gfpt siRNA+lanes), better seen on α-Fct WB. Right panel shows that, upon suppression of F, HA-F can fully complement for VP production, as no decrease in VP is observed (HA-F/Fgfpt lanes). (TIF) [file pone.0078074.s002.tif]

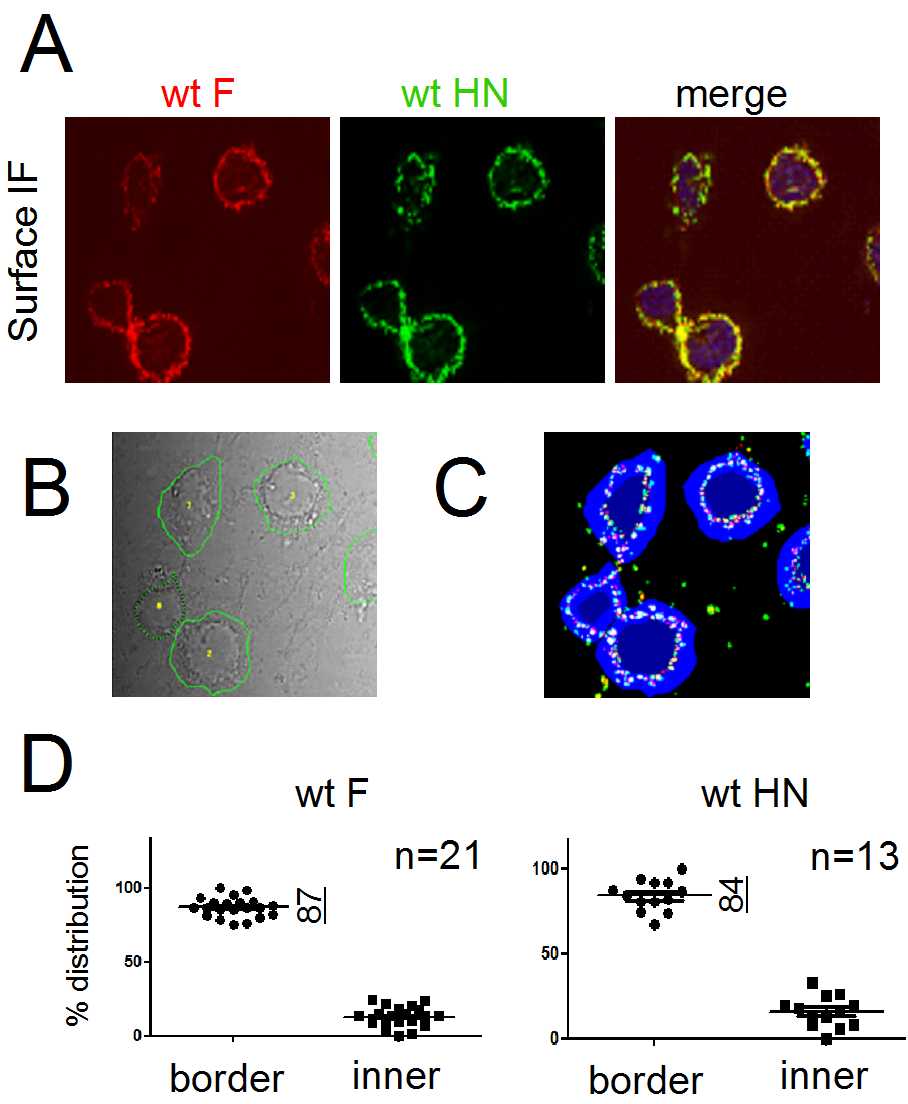

Supplement: Figure S3 — Statistical analysis of the distribution SeV envelope proteins in infected cells. A. MDCK cells constitutively expressing α-gfpt siRNA were grown on coverslips and infected with rSeV-HA-F/Fgfpt. Twenty-four hours post infection, cells were submitted to surface immunofluorescence (Surface IF) protocols, staining wt HA-F and HN. B. The stack of visible light images was processed to obtain the best focus for basic delimitation of cell borders. On the resulting images, cell outlines were provided manually and transferred to images of F and HN staining (merge in A). From these outlines, a cell border zone was delimited by dilatation to +/−10 pixels inside and outside. C. Defining the cell border (light blue) allowed definition of a cell inner compartment (dark blue). From each compartment, an event (corresponding to the presence of a given protein, F/HN/M), was identified by a signal having a minimal intensity level. D. Events were finally counted from both compartments and percentage of the protein distribution was calculated and presented graphically. As in this case one deals with surface immunofluorescence images of wt F and HN, the results serve as validation for the analysis approach as >85% of the staining is scored as expected in the border compartment. Graphical representation of the data was executed by Graph Pad Prism 6. Underlined vertical numbers = numerical averages. (TIF) [file pone.0078074.s003.tif]

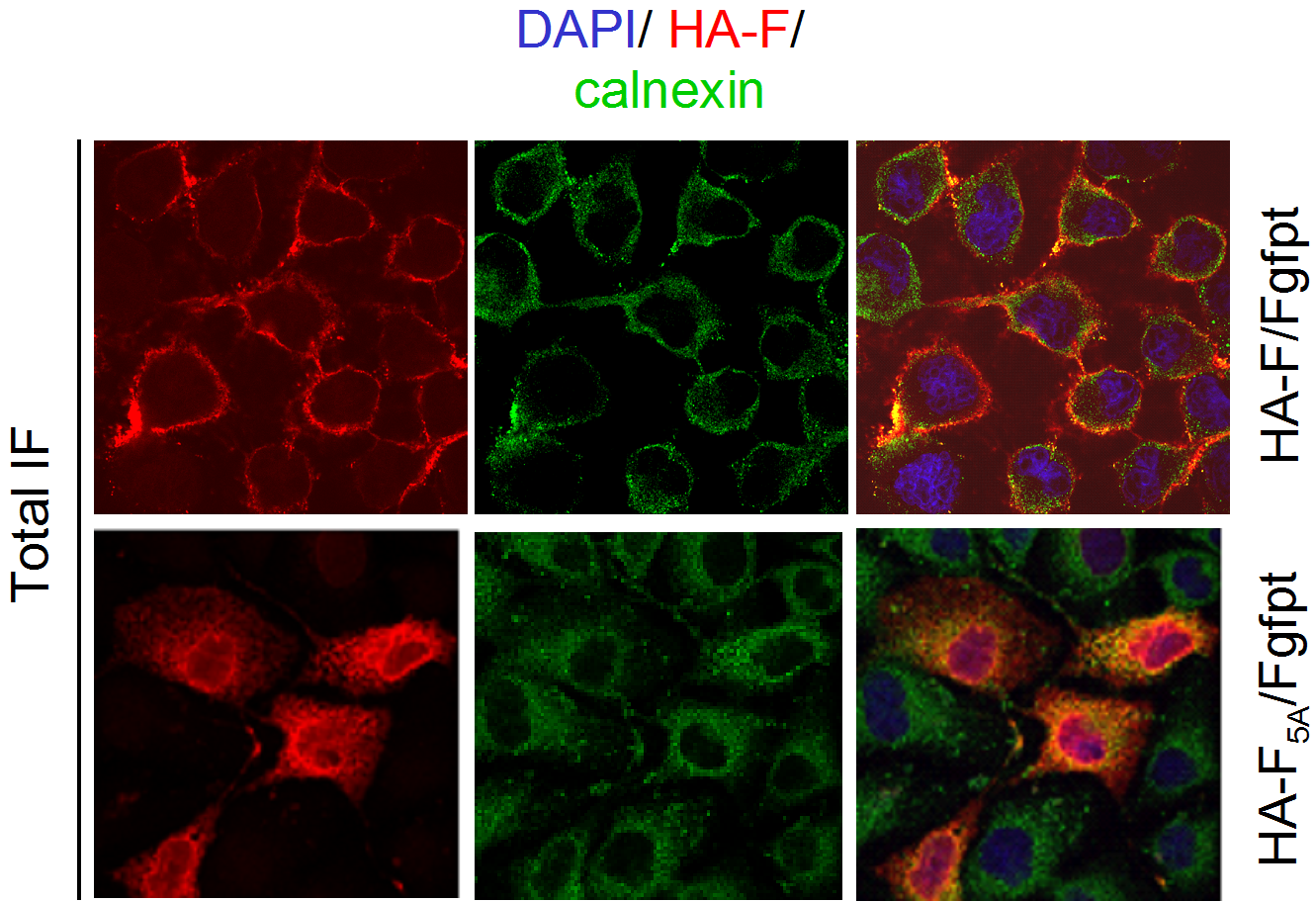

Supplement: Figure S4 — Subcellular localization of the wt HA-F and HA-F5A in the context of infections. The merge images of figure 5C are presented to visualize the prominent peri-nuclear staining of HA-F5A where it co-localizes with calnexin. This contrasts with the ring shape staining of HA-F at the border of the cell. (TIF) [file pone.0078074.s004.tif]

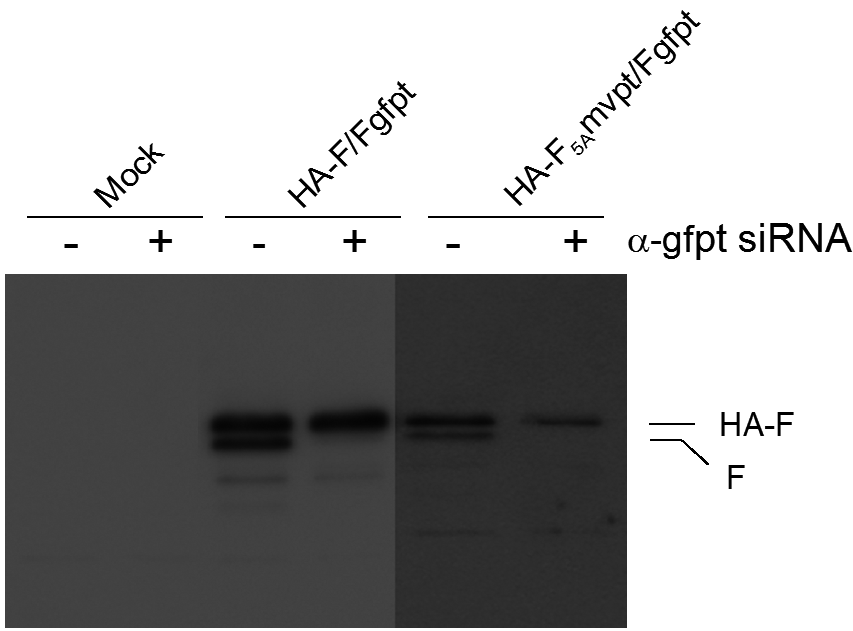

Supplement: Figure S5 — Inputs of the co-immunoprecipitations shown in Figure 5B . Western blot analysis of the MDCK cellular extract samples used to perform the co-IP presented in figure 4B. The samples were probed with α-Fct to visualize both HA-F and F so to appreciate the ratio of the two proteins in each extract. Quantitatively in excess to the HA-F5Amvpt/Fgfpt, the HA-F/Fgfpt samples were revealed with a shorter exposure, although all the samples were part of the same PAGE. (TIF) [file pone.0078074.s005.tif]

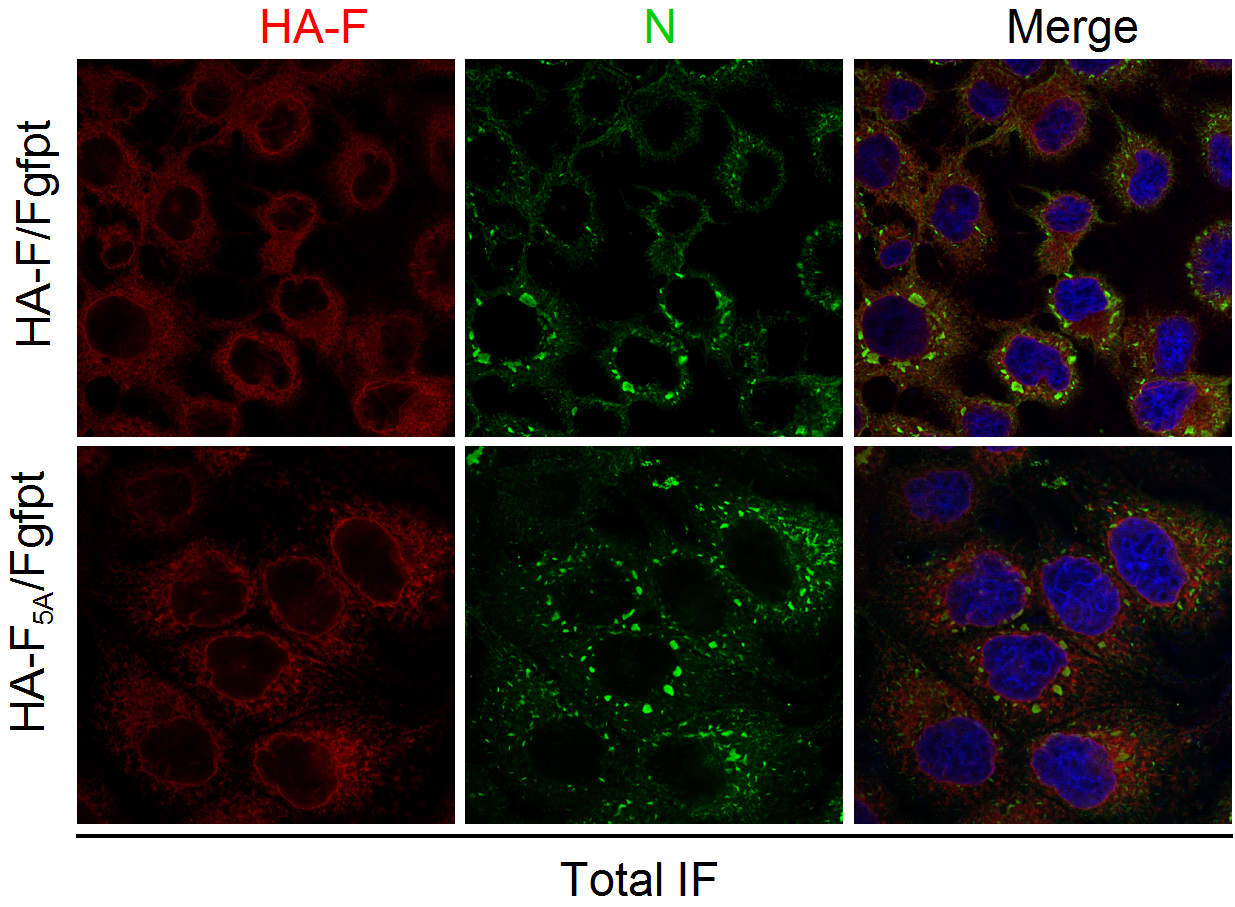

Supplement: Figure S6 — Subcellular localisation of N is not affected by presence or absence of F at the plasma membrane. MDCK cells constitutively expressing α-gfpt siRNA were grown on coverslips and infected with rSeV-HA-F/Fgfpt and rSeV-HA-F5Amvpt/Fgfpt. Twenty-four hours post infection, cells were submitted to total immunofluorescence protocol. α-HA and α-N were applied as primary antibodies to visualize HA-F and N protein, respectively. (TIF) [file pone.0078074.s006.tif]

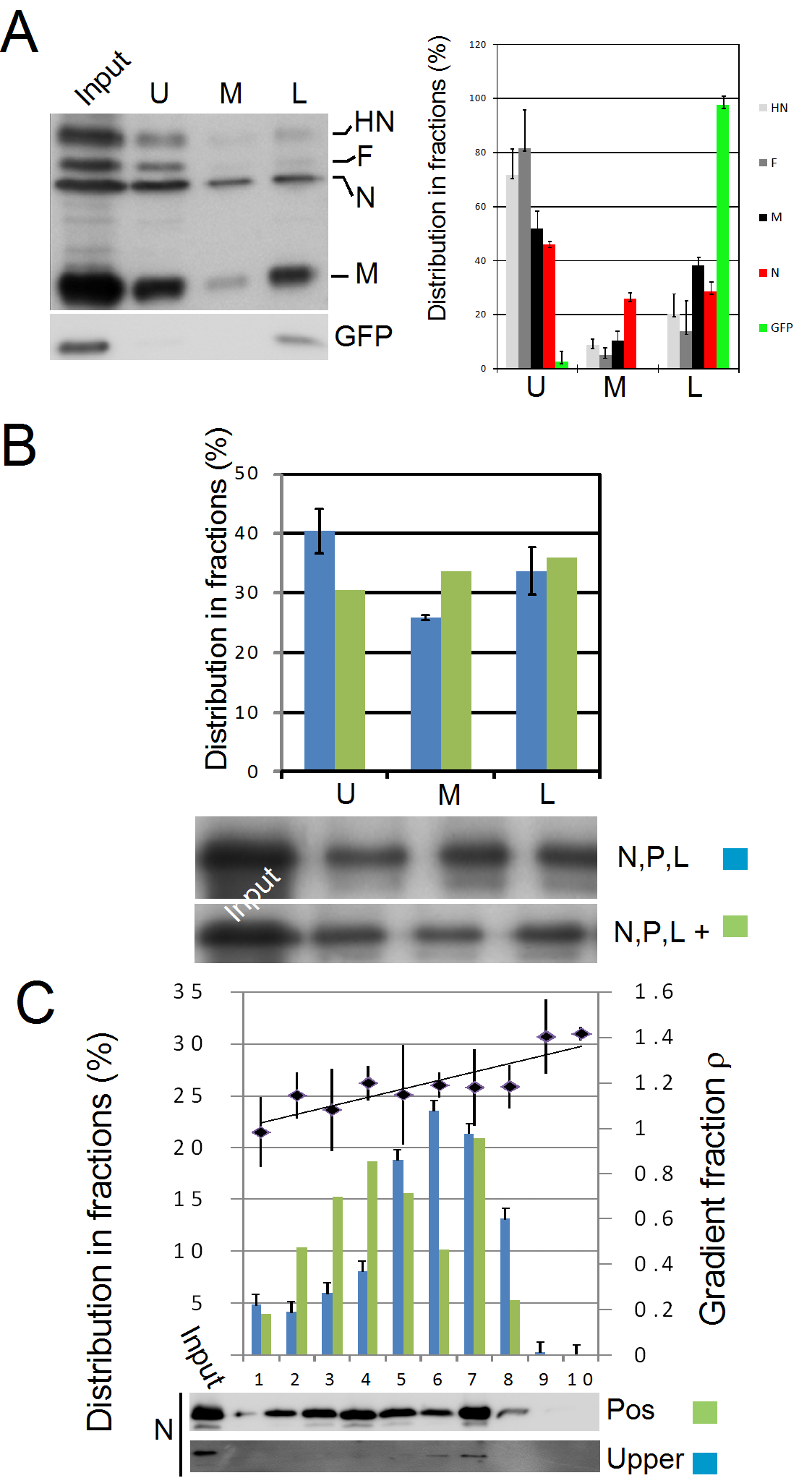

Supplement: Figure S7 — Part of SeV N associated with cellular membrane is in the form of nucleocapsid. A. rSeV-GFP infected MDCK cells, collected at 30 hours post-infection in 300 µl TNE (Tris-HCl pH 7.5 10 mM, NaCl 50 Mm mM, EDTA 1 mM) −10% sucrose, were disrupted by vortexing with 150 µl of glass beads. The cell lysate (300 µl) were mixed with 900 µl of 90% sucrose in TNE and set at the bottom of SW60.1 Beckman centrifuge tubes (50 µl was set aside representing the input). The floatation gradient was completed with 2.5 ml of sucrose 65% and finally 1 ml of 10% sucrose in TNE. After 12 hours of centrifugation at 12°C, 40 K, three 1.5 ml fractions were carefully collected from the top of the tubes, representing the upper (U), middle (M) and lower (L) samples. Fractions of these samples (50 µl) were mixed with 25 µl of 3× concentrated PAGE sample buffer, boiled and analysed by Western blots using a cocktail of μ-HN, F, N, M antibodies, plus α-GFP; GFP serving as the control protein not associated with membrane. Histogram at right: quantification of HN (light grey bar), F (dark grey), M (black), N (red) and GFP (green) protein fractions from 2 independent experiments. B. The N, P and L proteins were expressed from plasmids alone (upper western blot N,P,L; blue bars in histogram) or co-expressed with expression from plasmid of the full-length SeV RNA genome (lower western blot N,P,L +; green bars in histogram) in BSRT7 cells. Cell lysates were then analysed by floatation gradients as in (A). Histogram: quantification of two independent experiments. C. rSeV-GFP infected MDCK cell lysates prepared by disruption in 0.6% NP40, 50 mM Tris-HCl pH 7.0, 10 mM NaCl (lysing buffer I, [38] or Upper fraction obtained from N, P, L expressing BSRT7 cell floatation gradient analysis as presented in (B. N,P,L) were loaded onto linear 20–40% CsCl gradients and centrifuged for 2 hours (12°C, SW 41, 36 K, [38]. Nine 1.3 ml fractions were collected and analysed by Western blots using α-NSDS and density ρ of the [file pone.0078074.s007.tif]
